# Tuberculosis Transmission among Immigrants and Autochthonous Populations of the Eastern Province of Saudi Arabia

**DOI:** 10.1371/journal.pone.0077635

**Published:** 2013-10-17

**Authors:** Bright Varghese, Philip Supply, Mohammed Shoukri, Caroline Allix-Beguec, Ziad Memish, Naila Abuljadayel, Raafat Al-Hakeem, Fahad AlRabiah, Sahal Al-Hajoj

**Affiliations:** 1 Department of Infection and Immunity, King Faisal Specialist Hospital and Research Centre, Riyadh, Saudi Arabia; 2 CNRS UMR 8204, Lille, France; 3 INSERM, U1019, Lille, France; 4 Center for Infection and Immunity of Lille, Institut Pasteur de Lille, Lille, France; 5 University Lille Nord de France, Lille, France; 6 Centre for Biotechnology, King Faisal Specialist Hospital and Research Centre, Riyadh, Saudi Arabia; 7 Genoscreen, Lille, France; 8 Public Health Directorate, Ministry of Health, Riyadh, Saudi Arabia; 9 Department of Medicine, King Faisal Specialist Hospital and Research Centre, Riyadh, Saudi Arabia; Institut de Pharmacologie et de Biologie Structurale, France

## Abstract

**Background:**

Eastern province of Saudi Arabia is an industrial zone with large immigrant population and high level of tuberculosis case notification among immigrants. The impact of immigration and current trends of tuberculosis transmission among immigrants and autochthonous population in the region had not been investigated so far using molecular tools.

**Methodology:**

During 2009- 2011, a total of 524 *Mycobacterium tuberculosis* isolates were collected from the central tuberculosis reference laboratory, representing an estimated 79.2% of the culture-positive tuberculosis cases over the study period in the province. These isolates were genotyped by using 24 locus-based MIRU-VNTR typing and spoligotyping followed by first line drug susceptibility testing. The molecular clustering profiles and phylogenetic diversity of isolates were determined and compared to the geographical origins of the patients.

**Principle Findings:**

Genotyping showed an overall predominance of Delhi/CAS (29.4%), EAI (23.8%) and Ghana (13.3%) lineages, with slightly higher proportions of Delhi/CAS among autochthonous population (33.3 %) and EAI (30.9%) among immigrants. Rate of any drug resistance was 20.2% with 2.5% of multi-drug resistance. Strain cluster analysis indicated 42 clusters comprising 210 isolates, resulting in a calculated recent transmission index of 32.1%. Overall shared cluster ratio was 78.6% while 75.8% were shared between autochthonous population and immigrant population with a predominance of immigrants from South east Asia (40.7%). In contrast, cross national transmission within the immigrant population was limited (24.2%). Younger age (15-30- *p* value-0.043, 16-45, p value 0.030), Saudi nationality (*p* value-0.004) and South East Asian origin (*p* value-0.011) were identified as significant predisposing factors for molecular strain clustering.

**Conclusions:**

The high proportion of molecular clusters shared among the autochthonous and immigrant populations suggests a high permeability of tuberculosis transmission between both populations in the province. These results prompt for the need to strengthen the current tuberculosis control strategies and surveillance programs.

## Introduction

Tuberculosis (TB) is a reemerging infectious disease and a substantial public health problem globally despite the improvements in treatment and control [1]{Organization, 2012 #259;Organization, 2012 #259}. Increased movements of human populations tend to globalize the problem, by favoring transmission of disease cases from high-prevalence countries to low-prevalence regions. However the impact of such import is difficult to forecast, due to the chronic nature of TB, the long period before reactivation of remote infection and supposedly low levels of TB transmission [2]. This impact is also problematic to determine because of the difficulty to obtain representative mycobacterial strain collections, especially in a large country. Recent findings showed the association between the geographical origins of the patient’s genotypes of *Mycobacterium tuberculosis* and clinical manifestations of the disease [3-5]. Thus, capturing the impact of immigration on the population structure of the pathogen and its transmission among population groups in particular world regions is expected to help improving existing control strategies. 

In recent years the immigrant population in Saudi Arabia has markedly increased from 5.2 million (2000) to 8.97 million (2010) [6,7]. The national tuberculosis registry showed higher rate of TB incidence among immigrants (28 (in 2000)-26.7(in 2009) cases/100000 populations) compared to autochthonous population (11.7 (in 2000)-11.5(in 2009) cases/100000 populations) in the past few years [8,9]. Besides the possible transmission of TB among settled immigrants and the autochthonous population in the country, another possible factor is one of the largest human gathering (3.16 million pilgrims including more than one million of local pilgrims in 2012) in the world for a religious ritual “Holy Hajj” as recorded by different studies and authorities [10-12]. 

In the highly industrialized Eastern province, immigrant population elevated from 0.72 million in 2000 to 1.21 million in 2011 [13]. The previous policy of deportation of expatriate workers diagnosed with TB is still thought to be an impediment to health care seeking behavior of the considerable number of the illegal migrants particularly in this region [14]. Recurrent reports of the predominance of immigrants among annual TB case notifications and the new increasing trend of cases among young Saudi nationals in this province prompted us to explore, more in depth in this region, the possibilities of TB transmission between autochthonous and immigrant populations, recently suggested by our study based on a partial sample of drug-resistant strains at a national level [8,15-17]

Thus the study was designed to determine the lineages among the autochthonous and immigrant populations in this province, and measure the strain molecular clustering as a classical proxy to TB transmission between both populations, as well as evaluate possible risk factors for such clustering. 

## Materials and Methods

### Study population and setting

This study was reviewed and approved by the Research Ethics Committee of King Faisal Specialist Hospital and Research Centre. The study utilized only clinical isolates with anonymized data collection and analysis, hence a waiver for consent forms was provided. During July 2009 through June 2011, all the available non-repetitive *M. tuberculosis* complex isolates were enrolled into the study from the province. The isolate culturing and data collection were carried out in central tuberculosis reference laboratory of the Eastern province. This study utilized 77 drug resistant isolates collected from the province as part of another recently published nationwide study conducted at the same time [17]. The study population was divided into two major groups “Immigrants” and “Autochthonous” population (Saudi nationals, abbreviated as SA). Immigrants were defined as “individuals who born outside Saudi Arabia and holding a citizenship other than Saudi”. In addition to Saudi nationals born in the Kingdom, an assumed very minor fraction of individuals who were born outside of Saudi Arabia and later received the Saudi nationality were also considered as “Saudi/autochthonous” as their previous demographical data are usually not considered or available in the Ministry of Health records. The immigrant population was classified into four sub-groups according to their geographical origin as, African (AFR-Sudan, Eritrea, Somalia, Ethiopia, Egypt, Chad, Nigeria, Senegal, Cameroon), South Asian (SAS- India, Nepal, Sri Lanka, Bangladesh, Pakistan, Myanmar, Afghanistan), South East Asian (SEA- Philippines, Indonesia, Thailand and China) and West Asian (WA-Yemen). 

### Laboratory Procedures

The genomic DNA from heat denatured culture isolates were prepared by using standard spin column technique [QIAamp DNA mini kit, Qiagen, GmbH, Germany] according to the manufacturer’s instructions. All the isolates were subjected to identification as *M. tuberculosis* complex using the Genotype MTBC assay kit [Hain Life Science, Nehren, Germany]. First line drug susceptibility testing was conducted by using the BACTEC MGIT SIRE kit [Becton Dickinson, CA, USA]. The spoligotyping was carried out by using the commercially available membranes [Ocimum Biosolutions, Hyderabad, India] [18]. Standard 24-locus based MIRU-VNTR typing was performed with the commercial kit [Genoscreen, Lille, France] using 48 capillaries ABI 3730 genetic analyzer (Applied Biosystems, CA, USA) as described previously [19]. The MIRU-VNTR alleles were determined by using Genemapper V-4.0 (Applied Biosystems, CA, USA) and data were compiled by the MIRU-VNTR Data manager (Genoscreen, Lille, France).

### Data Analysis

The drug susceptibility results were classified as sensitive (no resistance), mono (resistance to a single drug), poly (resistance to more than one drug, other than both rifampicin and isoniazid) and multi drug resistant tuberculosis (MDRTB) (resistance to both rifampicin and isoniazid). The spoligotyping results were converted into octal codes. Mycobacterial genetic lineages were identified using MIRU-VNTR and spoligotyping data and the online tools available on MIRU-VNTR Plus and SITVIT web sites (www.miru-vntrplus.org & http://www.pasteur-guadeloupe.fr:8081/SITVIT_ONLINE/index.jsp) according to the previously described strategy, combining best-match and phylogenetic tree-based analysis [20,21]. Molecular clustering of the isolates was determined by constructing a dendogram based on spoligotyping and MIRU-VNTR data, using the unweighted pair group method using average linkages and the categorical coefficient. A strain cluster was defined as two or more isolates sharing completely identical fingerprints. The strain clustering rate was calculated by assuming that one patient from each strain cluster corresponded to the index case at the origin of infection as described in the previous study [22]. The recent transmission index (RTI)/strain clustering rate was calculated as,*RTIn*−1=(*nc*−*c*)/*n*. Where n_c_ is the total number of clustered cases, c is the number of clusters and n is the total number of cases in the study. Cross-national transmission was defined as the occurrence of a cluster comprising patients of at least two different nationalities. Isolates displaying double alleles in two or more VNTR loci suggestive of mixed genotypes, which could reflect either mixed infection or contamination, were excluded from analysis. For the analysis of the correlation of drug resistance vs lineage, we chose to regroup sub-lineages defined based on MIRU-VNTR and spoligo data into (larger) lineages as defined by Large Sequence Polymorphism (LSP) based classification of Gagneux et al [23] in order to have sufficiently large groups to enable statistical analysis. Correspondence between LSP and MIRU-VNTR-spoligotyping-based classification was respectively as follows: Indo-Oceanic lineage/EAI; East African Indian/Delhi/CAS; East Asian/Beijing and Euro-American/Haarlem, Ghana, LAM, S, Uganda-I and Cameroon respectively. The statistical analysis of the results was carried out by using the software package SPSS V-19.0 (IBM, USA). The association of five predisposing risk factors namely age, gender, infection type, drug resistance, and geographical origin against the strain clustering were studied. Chi square test was conducted to determine the statistical difference along with Bonferonni’s correction on p values. The *p* value <0.05 was considered as statistically significant. 

## Results

### Study Population

During the study period a total of 532 non-repetitive culture isolates were enrolled. This strain collection represented an estimated 79.2 % of the culture-positive tuberculosis cases over the study period in the province, thus virtually reaching the 80% threshold classically considered for defining a population-based study. Among the 532 isolates, 8 isolates were removed from the analysis as they were identified as *Mycobacterium bovis* by the line probe assay and further identified as BCG vaccine strains by the MIRU typing. 

**Figure 1:**
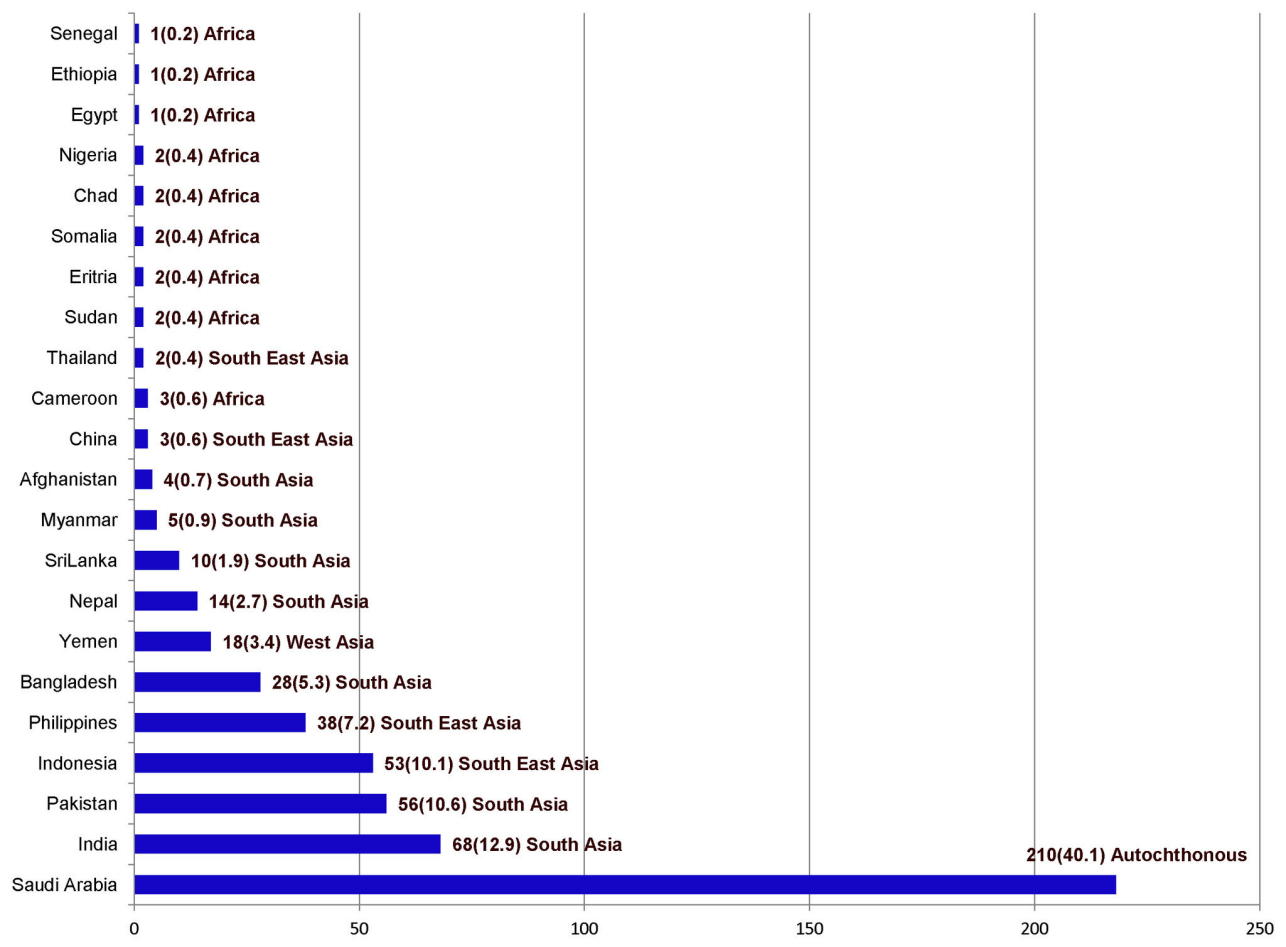
Geographical origin of the 524 study subjects. Figure shows the number of isolates enrolled into the study with nationality of the patients in the left side and number of cases with percentage of overall distribution and geographical origin in the right side.

Of the total enrolled cases, 40.1% were autochthonous and 59.9% were immigrant patients. The immigrant population consisted of individuals from 21 different countries, of which the most represented ones were India (12.9%), Pakistan (10.6%), Indonesia (10.1%), Philippines (7.2%) and Bangladesh (5.3%). The sub grouping of the 59.9 % immigrant cases according to the geographical origin was as follows: SAS (35.3%), SEA (18.3%), WA (3.2%) and AFR (3.1%) (Figure 1). 

Most patients were belonged to age groups of 15-30 (46.6%) and 31-45 (32.4%) years. However, patients aged <15 years and >60 years were mainly confined to the autochthonous population (100% and 78.3%, respectively). A domination of male patients (70.3%) was observed with a male/female (M/F) ratio of 2.4. Interestingly, two study groups, SEA and AFR appeared as exceptions by showing larger proportion of female cases (M/F ratios-0.71 and 0.66 respectively), supposedly mirroring an overrepresentation of women among these specific immigrant groups. Of the total, 389 cases (74.2%) were pulmonary TB, while 135 (25.8%) were extra pulmonary TB cases. Pulmonary TB dominated among the immigrant study groups with more than 72% (P/E ratio-2.7-7.5, depending upon the geographical origins) of the reported cases whereas extra pulmonary cases were relatively more frequent (32.8%) among Saudi population (Table 1). 

**Table 1:** Demographical summary of 524 cases enrolled into the study.

Parameters	Patient Geographical Origin (n/%)				
	Saudi (n=210)	South Asia (n=185)	South East Asia (n=96)	Africa (n=16)	West Asia (n=17)
Age Group					
**0-14**	4 (1.9)				
**15-30**	88(41.9)	83 (44.9 )	49 (51)	11 (68.7)	13 (76.4)
**31-45**	64 (30.5)	72 (41.6)	30 (33.3)	2 (12.5)	2 (11.8)
**46-59**	36 (17.1)	25 (10.8)	17 (15.6)	3 (18.7)	2 (11.8)
**>60**	18 (8.6)	5 (2.7 )			
Gender					
**Male**	138 (65.7)	169 (91.4)	40 (41.7)	5 (31.2)	14 (77.8)
**Female**	72 (34.3)	16 (8.6)	56(58.3)	11(68.8)	3 (22.2)
**M/F Ratio^1^**	1.9	10.6	0.71	0.66	3.5
Infection Type					
**Pulmonary**	141(67.2)	149 (80.5)	70 (72.9)	14 (87.5)	15 (88.2)
**Male**	101	139	33	5	13
**Female**	40	10	37	9	2
**Extra-pulmonary**	69 (32.8)	36 (19.5)	26 (27.1)	2 (12.5)	2 (11.8)
**Male**	32	30	7		1
**Female**	37	6	19	2	1
**P/E Ratio^2^**	2.0	4.1	2.7	7	7.5

1 Male/Female ratio

2 Pulmonary/extra-pulmonary ratio

### Distribution of phylogeographical lineages

Genotyping identified a total of 13 lineages, dominated overall by Delhi/CAS (29.4%), EAI (23.8%), Ghana (13.3%) and Haarlem (8.6%) lineages (Dataset S1). The isolates from autochthonous population showed a predominance of Delhi/CAS (33.3%) followed by Ghana (14.3%) and EAI (13.3%). On the other hand, the immigrant cases showed a prevalence of EAI (30.9%), followed by Delhi/CAS (26.7%) and Ghana (12.7%) lineages respectively. The lineage Beijing was found to be 8.6 % and 5.2 % among immigrant and Saudi cases, respectively. Noticeably, the lineage TUR and undefined strains were found only among Saudi nationals (Table 2). 

**Table 2:** Diversity and distribution of lineages according to the geographical origin of patients.

Lineages^1^	Study Groups (Case/Percentage)			Geographical origin of immigrants			
	Total (N=524)	Saudi (N=210)	Non-Saudi (N=314)	South Asia	South East Asia	Africa	West Asia
Delhi/CAS	154 (29.4)	70 (33.3)	84 (26.7)	71	10	2	1
EAI	125 (23.8)	28 (13.3)	97 (30.9)	52	39	4	2
Ghana	70 (13.3)	30 (14.3)	40 (12.7)	19	7	3	11
Haarlem	45 (8.6)	24 (11.4)	21 (6.7)	9	12		
Beijing	38 (7.2)	11 (5.2)	27(8.6)	12	14	1	
LAM	26 (5.0)	15 (7.1)	11 (3.5)	4	5	1	1
S	17 (3.2)	9 (4.3)	8 (2.6)	5	3		
Uganda-I	12 (2.3)	9 (4.3)	3 (1.0)	2	1		
URAL	11 (2.1)	6 (2.8)	5 (1.6)	3	1	1	
New-I	10 (1.9)	2 (0.9)	8 (2.5)	5	3		
X	7 (1.3)	1 (0.5)	6 (1.9)	3	1	2	
Cameroon	4 (0.8)		4 (1.3)			2	2
TUR	3 (0.6)	3 (1.4)					
Unknown	2 (0.4)	2 (0.9)					

1 Lineages assigned based on the MIRU-VNTR plus online database, number of lineages among the study group Non-Saudi showed as numbers.

### Drug resistance in the study

The overall frequency of drug resistance among the isolates was 20.2%, comprising 14.1%, 3.6% and 2.5% of mono, poly-drug resistance and MDRTB, respectively. The rate of drug resistance was slightly and not significantly higher among immigrants (21.9%) than Saudis (17.6%). Although drug resistance was seen among all the lineages, the isolates of the Euro-American super-lineage (50%) were more often resistant than those of the East African Indian (23.6%) and Indo-Oceanic (17.9%) lineages. Of the 13 MDRTB cases, 6 (46.1%) of them belonged to Euro-American lineages followed by 4 (30.8%) East Asian lineages and 3 (23.1%) Indo-Oceanic lineages (Table 3).

**Table 3:** Pattern of drug resistance in the study.

Parameters	Drug resistance pattern^1^			Total (N/%)
	Mono drug	Polydrug	MDRTB	
**Patient Origin**				
Saudi	23	5	9	37(17.6)
Non Saudi	51	14	4	69(21.9)
Total	74 (14.1)	19 (3.6)	13 (2.5)	106 (20.2)
P value	0.115	0.313	0.060	
**Lineages** ^2^				
Indo-Oceanic	15	1	3	19(16.7)
East African Indian	18	7	-	25 (21.9)
East Asian	5	-	4	9 (7.9)
Euro-American	36	11	6	53 (46.5)
P value	0.509	0.141	0.005	

1 Drugs used for susceptibility testing, Streptomycin, Isoniazid, Rifampicin and Ethambutol, poly-resistance- resistance to more than one drug, MDRTB- resistance to both INH and RIF

2 Lineages found in this study are defined as, Indo-Oceanic lineage- EAI; East African Indian-CAS (Delhi/CAS); East Asian-Beijing; Euro-American–Haarlem, Ghana, LAM, S, Uganda-I and Cameroon.

### Molecular cluster analysis

Molecular cluster analysis based on MIRU-VNTR allele types and spoligotypes identified 356 distinct genotypes including 42 in clusters comprising from 2 to 24 isolates each and a total of 210 isolates, while 312 types were unique. On the other hand spoligotyping alone obtained 63 types, of which 25 were found in clusters with a size of 2-54 isolates and 38 were orphans. The calculated recent transmission index (clustering rate) in the study was 32.1%. Nine lineages were found among the clustered isolates with high cluster rates, majorly LAM (53.8%), Ghana (48.6%), S (47.1%) and EAI (43.2%) (Table 4). Figure 1 shows the lineage distribution among the 210 clustered isolates. 

**Table 4:** Nomenclature of strain clusters and their distribution among patients of different nationalities.

MIRU Lineage^1^	SIT^2^	Clades	Clustered isolates	Immigrants (N/%)	Nationalities
EAI	1501	EAI-2 Manila	5	3(60)	Philippines, Indonesia, SA^3^
EAI	1501	EAI-2 Manila	8	8(100)	Philippines, Indonesia, India
EAI	109	EAI-8 MDG	2		SA
EAI	11	EAI_3 India	4	4(100)	India, Bangladesh
EAI	11	EAI_3 India	2	2(100)	Sri Lanka
EAI	654	EAI_3 India	2	2(100)	Bangladesh
EAI	299	EAI_5	2	2(100)	Somalia
EAI	19	EAI_2 Manila	4	2(50)	Philippines, Indonesia, SA
EAI	19	EAI_2 Manila	4	1(25)	Indonesia, SA
EAI	19	EAI_2 Manila	21	21(100)	Philippines
Haarlem	47	H1	6	2(33.3)	Pakistan, Indonesia, SA
Haarlem	47	H1	5	3(60)	Bangladesh, Philippines, SA
Haarlem	47	H1	3	1(33.3)	Indonesia, SA
Haarlem	50	H1	3		SA
Haarlem	168	H3	2	2(100)	Indonesia, Nepal
URAL	35	H3	4	3(75)	Indonesia, Philippines, SA
Ghana	53	T1	15	9(60)	Yemen, Nigeria, Chad, Cameroon, SA
Ghana	53	T1	8	3(50)	Yemen, SA
Ghana	53	T1	6	4(66.7)	Yemen, SA
Ghana	53	T1	5	3(60)	Yemen, Cameroon, SA
LAM	42	LAM-9	3	2(66.7)	Pakistan, SA
LAM	162	LAM-9	8	3(37.5)	Indonesia, SA
LAM	162	LAM-9	3	1(33.3)	Philippines, SA
Beijing	1	Beijing	3	1(33.3)	Indonesia, SA
Beijing	255	Beijing	6	6(100)	Indonesia, China, Thailand
Beijing	1	Beijing	4	2(50)	Philippines, SA
Delhi/CAS	1401	CAS1_Delhi	5	5(100)	Bangladesh, Nepal
Delhi/CAS	1401	CAS1_Delhi	5	5(100)	Bangladesh, India, Pakistan
Delhi/CAS	1401	CAS1_Delhi	3	1(33.3)	Bangladesh, SA
Delhi/CAS	26	CAS1_Delhi	6	5(83.3)	Bangladesh, India, SA
Delhi/CAS	26	CAS1_Delhi	9	6(66.7)	Bangladesh, India, Nepal, Pakistan, SA
Delhi/CAS	26	CAS1_Delhi	7	7(100)	Bangladesh, India, Myanmar, Nepal
Delhi/CAS	486	CAS1_Delhi	4	2(50)	Pakistan, SA
Delhi/CAS	357	CAS1_Delhi	2	1(50)	India, SA
Delhi/CAS	25	CAS1_Delhi	2	2(100)	Bangladesh
Delhi/CAS	25	CAS1_Delhi	9	7(77.8)	Bangladesh, India, Nepal, Pakistan, SA
Delhi/CAS	25	CAS1_Delhi	3	3(100)	Pakistan, Afghanistan
Delhi/CAS	1264	CAS	2	2(100)	Pakistan
Delhi/CAS	1093	CAS	4	3(75)	India, Sudan, SA
S	784	S	3	1(33.3)	Indonesia, SA
S	34	S	5	2(40)	Philippines, SA
TUR	41	LAM7-TUR	3		SA

1 Lineage defined using MIRUVNTR Plus online database

2 Defined with the help of SITVIT web.

3 Saudi Arabia

### Cluster analysis against patient geographic origin

**Figure 2:**
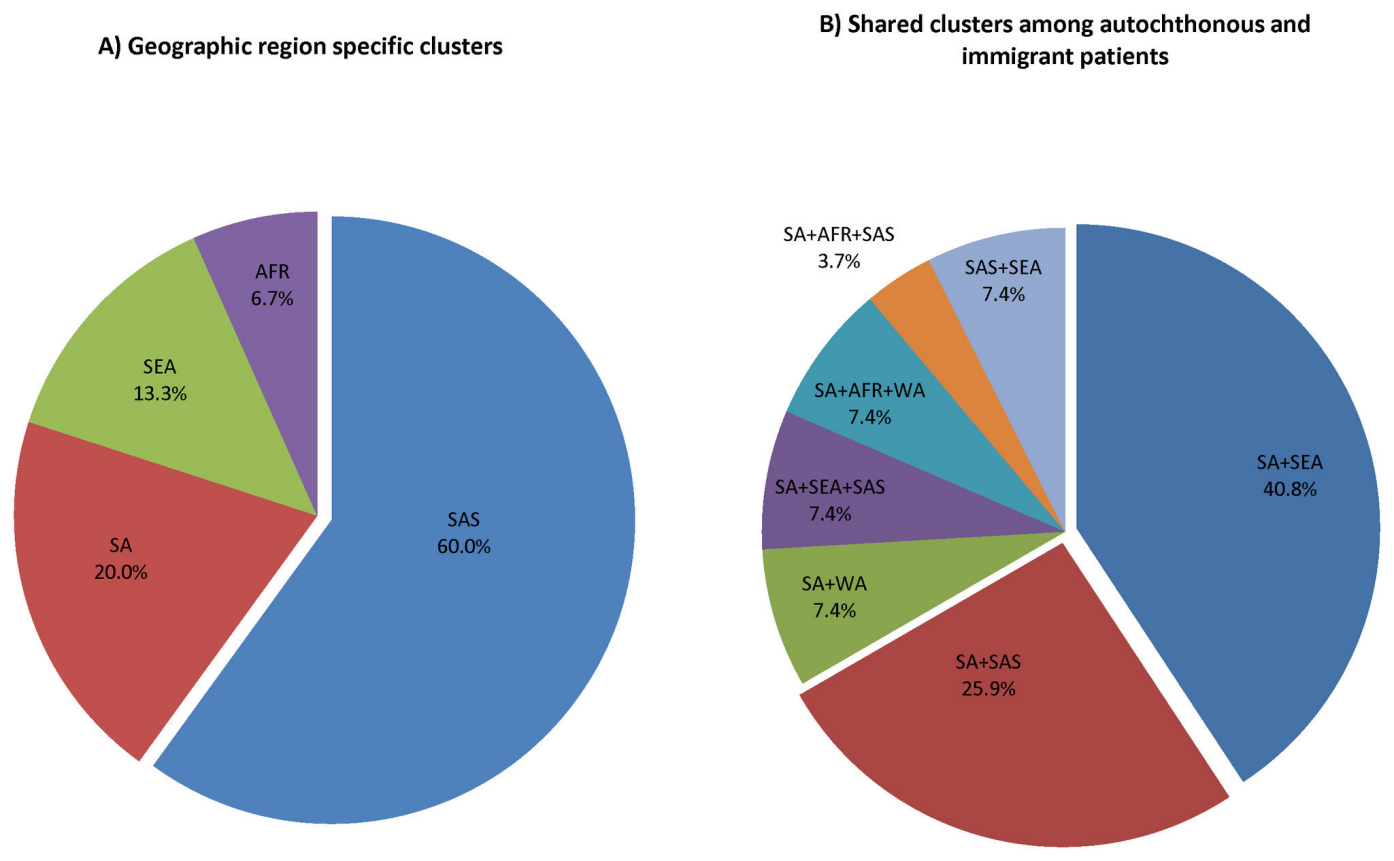
Strain cluster distribution among different study groups. Geographical origin of the patients’ versus number of strain clusters. Figure A shows the geographic region specific clusters found in the study. Figure B shows the different cross-national clustering between the immigrant and autochthonous population. The single cluster between immigrant study groups (SEA+SAS) was not shown in the figure.

A detailed cluster analysis over the different study groups was conducted. Analysis of strain clusters against the geographical origins of the patient showed that seventeen nationalities were represented among the total clustered isolates. Thirty-three clusters were shared between any of the study groups while 9 clusters were exclusively composed of patients belonging to a specific country. Such geographically homogenous clusters were prominently seen among the study group SAS (60%) followed by SA (20%) (Figure 2A). 

The three clusters exclusively corresponding to autochthonous patients, comprised EAI, Haarlem and TUR lineages respectively, while the nine clusters solely composed of SAS patients included strains of the Delhi/CAS and EAI lineages. In addition, the largest cluster (21 cases) in the study which was exclusively composed of Philippines nationals consistently corresponded to an EAI2-Manilla genotype (SIT 19) (Table 4).

Interestingly, overall rate of clusters shared between the study groups was 78.6%. On the other hand 75.8% (25 of 33 shared clusters) were shared between autochthonous and immigrant population. The majority of the mixed clusters were found between SA and SEA (40.8%) patients, followed by SA and SAS (25.9%) patients (Figure 2B). These mixed clusters corresponded to EAI, Haarlem, URAL, LAM, Beijing, S, Delhi/CAS and LAM strain genotypes, respectively (Table 4).

### Analysis of risk factors of strain clustering

Epidemiological parameters considered as plausible risk factors to be in a cluster, supposedly reflecting recent transmission, were analyzed. No statistical difference could be proven between males and females. However age group 15-30 (p value 0.043) and 31-45 (p value 0.030) showed a significant association with strain clustering. Saudi nationality (*p* value 0.004) and South East Asian origin (p value-0.011) were significantly associated to be in a strain cluster (Table 5). 

**Table 5:** Predisposing factors for molecular strain clustering in the province.

Risk Factors	Total cases	Clustered cases/% (N=210)	P value
**Age groups**			
0-14	4	3 (1.4)	-
15-30	244	86(40.9)	0.043
31-45	170	80(38.1)	0.030
46-59	83	35(16.6)	0.762
>60	23	6(2.8)	0.236
**Gender**			0.583
Male	366	150(71.4)	
Female	158	60(28.6)	
**Type of infection**			0.079
Pulmonary (Smear positive)	389	165(78.6)	
Extra pulmonary or pulmonary smear negative	135	45(21.4)	
**Drug susceptibility**			0.076
PAN susceptible^1^	418	176(83.8)	
Drug resistant	10	34(16.2)	
**Geographical origin**			
Saudi	210	68(32.4)	0.004
South Asia	185	77(36.6)	0.659
South East Asia	96	50(23.8)	0.011
West Asia	17	8(3.8)	0.729
Africa	16	7(3.3)	0.963

1 Susceptible to all the first line drugs tested.

## Discussion

This population based cross-sectional study was designed to evaluate the impact of the diverse human populations on the transmission and molecular epidemiology of tuberculosis in the Eastern province of Saudi Arabia. This province is well known for its relatively more liberal social mixing of immigrants and local population than any other county in Saudi Arabia. In this province, high TB notification rates are recurrently reported among the immigrants [14]. Consistently, the immigrant study population belonged to 21 countries of origin, most of which were notified as TB endemic areas by WHO with an annual prevalence ranging from 85 to 520 cases/100000 populations [1]. 

Male gender domination (70.3%) in the study, resulting male/female (M/F) ratio of 2.4 was similar to another recent report [24]. This may probably reflect as well in this specific case the domination of the male (0.93 million) compared to the female immigrant population (0.28 million) in the province. Analysis of drug resistance rate between immigrants and autochthonous population showed no significant differences. Thus it can be assumed that the drug resistances are not always "imported" in this province. Supportively ongoing transmission of drug resistance among immigrants was reported in recent studies [17,25]. 

A recurrent theme in Western countries receiving migrants is usually distinct distribution of *M. tuberculosis* strain lineages between the migrant and the autochthonous populations [23,26-29] and the often relatively low proportion of cross-national strain molecular clusters between locally-born and foreign-born patients [24,30-35] with a few exceptions [36]. In contrast, the distribution of phylogenetic lineages among the local population and immigrants were highly admixed in our study. Apart from two lineages (Cameroon only found among immigrants, and TUR only found among autochthonous populations), the rest of the 11 major lineages were found in both the immigrant and autochthonous populations (Table 2). 

Along the same lines, a substantial proportion of the molecular clusters were shared among the immigrant and autochthonous populations. While the strain clustering was higher among the immigrants (45.2%) compared to the autochthonous population (32.4 %), 25 of the 42 clusters identified in total included both migrant and autochthonous patients. The current findings most likely reflect the higher daily social admixing between both patient groups compared to Western countries, and its impact on the tuberculosis epidemiology in Saudi Arabia. Consistently with this proposal, the study group SEA (40.8%) showed the highest number of shared clusters with the autochthonous population followed by SAS (25.9%). As a reflection, Saudi (*p* value 0.004) and SEA (*p* value 0.011) geographic origins showed significant association with strain molecular clustering. The dominance of the study group SEA is coherent with the considerable population of workers from Indonesia and Philippines in the household who are in constant contact with the Saudi families. Supportively, among the 46 patient isolates in the 11 clusters of SA+SEA, 30 were from Saudi nationals and the remaining 16 were from SEA. Available information showed that 9 of these 16 immigrants were working either as house drivers or housemaids for some of the SA patients in same clusters. Although further occupational data of the enrolled subjects were not available for analysis, these data tend to indicate that transmission of TB in the households is a major factor favoring permeability of TB transmission between autochthonous populations and immigrant populations in the province. 

Of the 42 clusters in total, 9 were exclusively nationality-specific. Of these, three comprised solely autochthonous patients. One of these comprised TUR isolates. The fact that no TUR strains were found among any immigrant patient (either as unique strains or in other clusters) added to the geographic distribution of TUR strains (centered on Turkey, close to the study region) is suggestive of local transmission among autochthonous patients of a clone of local origin. However, the low number of TUR isolates identified overall makes this conclusion uncertain. The other six clusters comprised immigrants of same country of origin (eg. a cluster of EAI included two patients from Sri Lanka and another cluster of Delhi/CAS comprised two Pakistani patients) (Table 4). This finding is compatible with some degree of local transmission within groups of individuals with same nationality, assumed to be less frequent than transmission between groups of individuals with different nationalities in previous studies [37,38]. 

Along the same lines, the largest proportion (9 of 13) of clusters homogenously composed of patients from a same geographic region (i.e. different but neighboring countries) was interestingly seen in the study group SAS. This finding plausibly reflects the high number of non-skilled immigrant workers from countries like Pakistan, India, Nepal, Sri Lanka and Bangladesh who live in labor camps under crowded conditions and preferably sharing places with persons of same country of origin. Poor nutrition, lack of fresh air, stress and hardships may also favor reactivation of remote infection followed by transmission under such conditions. Added to that drug resistance might promote subsequent clinical manifestations also [39,40]. Unfortunately contact tracing data were not available to further assess the hypothesis of local transmission within this specific study group, or within e.g. the largest cluster caused by a SIT 19, EAI2-Manilla strain found only among Philippines nationals in the SEA group. 

Analysis of predisposing risk factors other than geographical origin (see above) for molecular strain clustering showed statistically significant association of age groups 15-30 (p value 0.043) and 31-45 (p value 0.030). The prominent role of younger age group as a possible risk factor in the study is in line with the large number of reported TB cases among young aged individuals in the country [8]. In addition the largest number of immigrants in the country consists of persons within the age group 15-45 and large cluster rates in this age group are in concordance with previous studies [16,41]. 

This study has certain limitations. Classical epidemiological contact investigation was not conducted in order to confirm TB transmission assumed from molecular clustering. Although population-based, the study may underestimate the recent transmission due to the short period of observation. Furthermore no screening experiments for extremely drug resistant isolates were carried out. 

## Conclusions

Nevertheless, we conclude that the socio-economical characteristics of the migrant group in our study population have a distinctive and detectable impact onto the molecular epidemiology and transmission of tuberculosis in the Eastern province of Saudi Arabia. The molecular evidence for cross-national transmission particularly between autochthonous and immigrant population gained from the present population-based study confirms and extends the observation obtained in our previous study, performed on a largely incomplete and only drug-resistant strain collection at a national level [17]. This evidence is likely reflecting the specific social admixing in the country. In addition, still large number of unique isolates among immigrants alone shows the possibility of reactivation of latent infection from their home country as evidenced in a recent study [25]. Combination of contact tracing and molecular analysis among high risk population will improve the management of targeted tuberculosis cases in a heterogeneous population. Further research with extended period (>5 years) of surveillance covering the whole nation using a highly detailed national registry (including immigration date information) and whole genome sequencing of *M. tuberculosis* isolates for investigation of clusters suggested by genotyping will reveal the real picture of transmission dynamics and the role of immigrants in the whole country. 

## Supporting Information

Figure S1 **Molecular clusters of *M. tuberculosis* isolates based on MIRUVNTR alleles and spoligo signatures.** The UPGMA tree was built and clusters were identified based on isolates sharing identical MIRU-VNTR types and spoligotypes.
(TIF)

Dataset S1 **MIRU-VNTR profiles of 524 enrolled cases.** The table shows the MIRU- VNTR alleles data and identified lineages of the enrolled study isolates.
(PDF)
