# Supplementary figures and images for: Tuberculosis Transmission among Immigrants and Autochthonous Populations of the Eastern Province of Saudi Arabia
Source: PLoS One. 2013 Oct 17;8(10):e77635. doi: 10.1371/journal.pone.0077635 (PMC3798324; doi:10.1371/journal.pone.0077635)

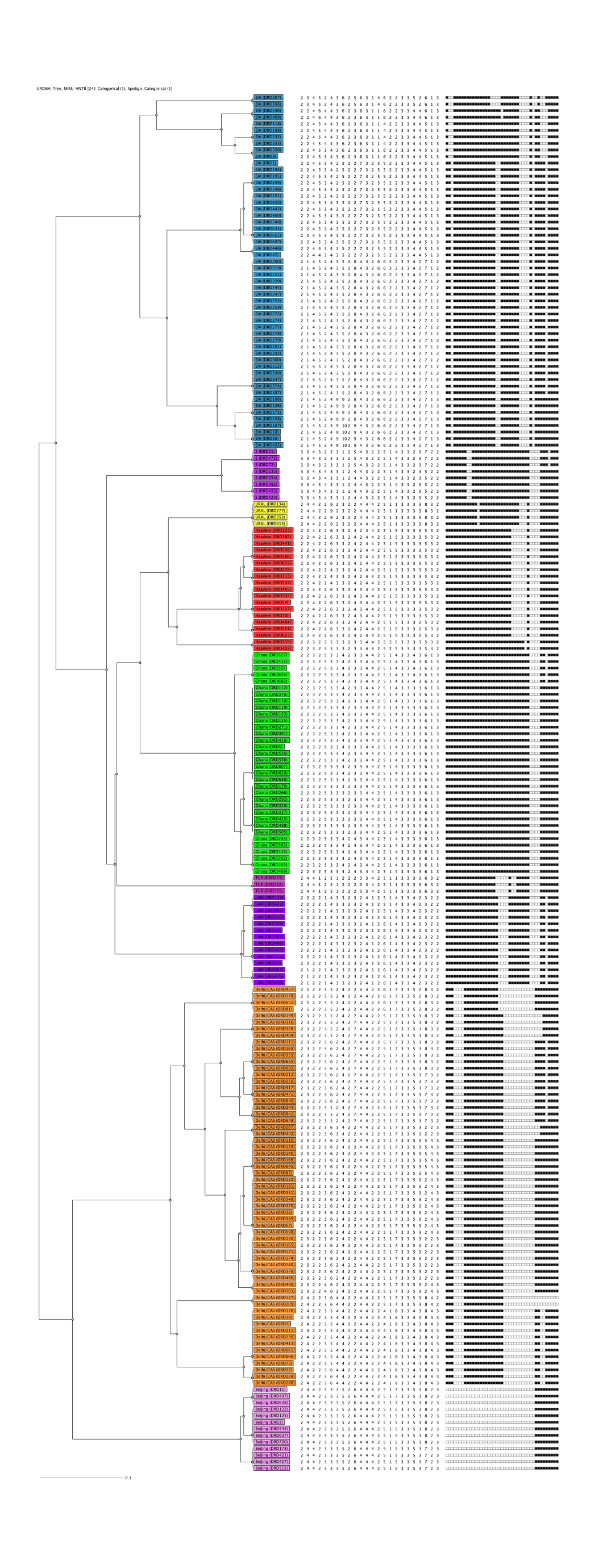

Supplement: Figure S1 — Molecular clusters of M. tuberculosis isolates based on MIRUVNTR alleles and spoligo signatures. The UPGMA tree was built and clusters were identified based on isolates sharing identical MIRU-VNTR types and spoligotypes. (TIF) [file pone.0077635.s001.tif]
