# Supplementary material for: Tuberculosis Transmission among Immigrants and Autochthonous Populations of the Eastern Province of Saudi Arabia
Source: PLoS One. 2013 Oct 17;8(10):e77635. doi: 10.1371/journal.pone.0077635 (PMC3798324; doi:10.1371/journal.pone.0077635)
Supplement: Dataset S1 — MIRU-VNTR profiles of 524 enrolled cases. The table shows the MIRU- VNTR alleles data and identified lineages of the enrolled study isolates. (PDF) [file pone.0077635.s002.pdf]

Sl No	ID	154	424	577	580	802	960	1644	1955	2059	2163b	2165	2347	2401	2461	2531	2687	2996	3007	3171	3192	3690	4052	4156	4348	Lineage
1	DRD11	2	4	4	2	3	3	3	5	2	6	4	4	4	2	5	1	7	3	3	5	3	8	2	3	Beijing
2	DRD113	2	4	4	2	3	3	3	3	2	6	4	4	4	1	5	1	7	3	3	5	3	8	2	3	Beijing
3	DRD122	2	4	4	2	3	3	3	5	2	6	4	4	4	2	5	1	5	3	3	5	3	8	2	3	Beijing
4	DRD125	2	4	4	2	3	3	3	5	2	6	4	4	4	2	5	1	5	3	3	5	3	8	2	3	Beijing
5	DRD139	2	4	4	2	3	3	3	6	2	3	4	4	4	2	5	1	7	3	3	5	3	7	2	3	Beijing
6	DRD172	2	4	4	2	3	2	3	5	2	6	4	4	4	2	5	1	5	3	3	5	3	8	2	3	Beijing
7	DRD178	2	4	4	2	3	3	3	5	2	3	4	4	4	2	5	1	5	3	3	5	3	8	2	3	Beijing
8	DRD193	2	4	4	2	3	2	3	5	1	6	3	4	4	2	5	1	5	3	3	5	3	7	2	3	Beijing
9	DRD223	2	4	4	2	3	2	3	5	2	6	3	4	4	2	5	1	5	3	3	5	3	8	2	3	Beijing
10	DRD276	2	4	4	2	3	3	3	6	2	3	4	4	4	1	5	1	5	3	3	5	3	8	2	3	Beijing
11	DRD295	2	4	4	2	3	2	3	5	2	3	3	4	4	2	5	1	7	2	3	5	3	7	2	3	Beijing
12	DRD297	2	4	4	2	3	2	3	5	2	3	3	4	4	2	5	1	7	3	3	5	3	7	2	3	Beijing
13	DRD3	2	4	4	2	3	3	3	5	2	6	4	4	4	2	5	1	5	3	3	5	3	8	2	3	Beijing
14	DRD309	2	4	4	2	3	3	3	6	2	6	4	4	4	2	5	1	7	3	3	5	3	7	2	3	Beijing
15	DRD33	2	4	4	2	3	3	4	6	2	3	4	4	4	2	5	1	5	3	3	5	3	8	2	3	Beijing
16	DRD334	2	4	4	2	3	2	3	6	2	3	4	4	4	2	5	1	5	3	3	5	3	8	2	3	Beijing
17	DRD418	2	4	4	2	3	3	3	5	2	6	4	4	4	2	5	1	7	3	3	5	3	7	2	3	Beijing
18	DRD421	2	4	4	2	3	3	3	5	2	3	4	4	4	2	5	1	5	3	3	5	3	8	2	3	Beijing
19	DRD427	2	4	4	2	3	3	3	5	2	3	4	4	4	2	5	1	5	3	3	5	3	8	2	3	Beijing
20	DRD459	2	4	4	2	3	3	3	6	2	6	4	4	4	2	5	1	4	3	3	5	3	8	2	3	Beijing
21	DRD461	2	4	4	2	3	2	3	5	2	3	3	4	4	2	5	1	7	3	3	5	3	8	2	3	Beijing
22	DRD494	2	4	4	2	3	3	3	5	2	3	4	4	4	2	5	1	5	3	3	5	3	7	2	3	Beijing
23	DRD497	2	4	4	2	3	3	3	5	2	6	4	4	4	2	5	1	7	3	3	5	3	8	2	3	Beijing
24	DRD506	2	4	4	2	3	3	3	5	2	3	4	4	4	1	5	1	5	3	3	5	3	7	2	3	Beijing
25	DRD515	2	4	4	2	3	3	3	5	2	3	4	4	4	2	5	1	5	3	3	5	3	8	2	3	Beijing
26	DRD520	2	4	4	2	3	2	2	4	2	3	3	4	4	2	5	1	5	3	3	5	3	7	2	3	Beijing
27	DRD527	2	3	3	2	3	3	2	5	2	3	4	4	4	2	5	1	5	3	3	5	3	7	2	3	Beijing
28	DRD544	2	4	4	2	3	3	3	5	2	6	4	4	4	2	5	1	5	3	3	5	3	8	2	3	Beijing
29	DRD551	2	4	4	2	3	2	3	5	2	3	3	4	4	2	5	1	5	3	3	5	3	7	2	3	Beijing
30	DRD585	2	2	4	2	3	3	3	6	2	3	4	4	4	2	5	1	5	3	3	5	3	8	2	3	Beijing
31	DRD637	2	4	4	2	3	3	3	5	2	6	4	4	4	2	5	1	5	3	3	5	3	8	2	3	Beijing
32	DRD638	2	4	4	2	3	3	3	5	2	6	4	4	4	2	5	1	7	3	3	5	3	8	2	3	Beijing
33	DRD674	2	4	4	2	3	3	3	5	2	3	4	4	4	2	5	1	7	3	3	2	3	8	2	3	Beijing
34	DRD700	2	4	4	2	3	3	3	5	2	6	4	4	4	2	5	1	5	3	3	5	3	8	2	3	Beijing
35	DRD701	2	4	4	2	3	3	3	6	2	6	4	4	4	2	5	1	5	3	3	4	3	8	2	3	Beijing
36	DRD785	2	4	4	2	3	1	3	5	2	3	3	4	4	2	5	1	7	2	3	5	3	7	2	3	Beijing
37	DRD87	2	4	4	2	3	2	3	5	2	3	4	4	4	2	5	1	5	3	3	5	3	7	2	3	Beijing
38	DRD895	2	4	4	2	3	2	3	5	2	3	3	4	3	2	5	1	7	2	3	5	3	7	2	3	Beijing
39	DRD105	2	2	4	2	3	4	3	3	1	6	3	4	2	2	5	1	5	3	3	3	7	5	2	2	Cameroon

40	DRD228	2	2	4	2	3	4	3	3	1	5	3	4	2	2	5	1	5	3	3	3	7	5	2	2	Cameroon
41	DRD249	2	2	4	2	3	4	3	3	1	4	3	4	2	2	5	1	5	3	3	3	7	5	2	2	Cameroon
42	DRD65	2	2	4	2	3	4	2	3	1	6	3	4	2	2	5	1	5	3	3	3	7	5	2	2	Cameroon
43	DRD111	2	3	2	2	3	6	2	4	2	7	4	4	2	2	5	1	7	3	3	5	3	8	3	2	Delhi/CAS
44	DRD116	2	3	2	2	3	6	2	4	2	2	4	4	2	2	5	1	7	3	3	5	3	5	4	3	Delhi/CAS
45	DRD120	2	5	2	2	3	5	2	4	2	2	4	4	2	2	6	1	7	3	3	4	2	7	3	2	Delhi/CAS
46	DRD123	2	3	2	2	3	6	2	4	2	2	3	4	2	2	5	1	7	3	3	5	3	2	2	3	Delhi/CAS
47	DRD129	2	3	2	2	3	6	2	4	2	2	4	4	2	2	5	1	7	3	3	5	3	5	4	3	Delhi/CAS
48	DRD130	2	3	2	2	3	6	2	4	2	2	4	4	2	2	5	1	7	3	3	5	3	2	2	3	Delhi/CAS
49	DRD132	2	3	2	2	3	6	2	4	2	2	4	4	2	2	5	1	7	3	3	5	3	2	4	3	Delhi/CAS
50	DRD140	2	3	2	2	3	6	2	4	2	2	4	4	2	2	5	1	7	3	3	5	3	5	4	3	Delhi/CAS
51	DRD149	2	3	2	2	3	5	2	4	2	7	3	4	2	2	5	1	4	3	3	5	3	8	3	2	Delhi/CAS
52	DRD15	2	4	2	2	3	4	4	5	2	2	4	4	2	2	5	1	7	3	3	4	3	2	4	2	Delhi/CAS
53	DRD165	2	3	2	2	3	6	2	4	2	2	4	4	2	2	5	1	7	3	3	5	3	2	2	3	Delhi/CAS
54	DRD166	2	3	2	2	3	6	2	4	2	2	4	4	2	2	5	1	7	3	3	5	3	5	4	3	Delhi/CAS
55	DRD167	2	4	2	2	3	7	4	4	2	2	4	4	2	2	5	1	6	3	3	5	3	5	4	3	Delhi/CAS
56	DRD169	2	3	2	2	3	6	2	4	2	7	4	4	2	2	5	1	7	3	3	5	3	8	3	2	Delhi/CAS
57	DRD17	2	3	2	2	3	6	2	4	2	2	4	4	2	1	5	1	7	3	3	5	3	2	4	3	Delhi/CAS
58	DRD171	2	3	2	2	3	6	2	4	2	2	4	4	2	2	5	1	7	3	3	5	3	2	2	3	Delhi/CAS
59	DRD174	2	3	2	2	3	6	2	4	2	2	4	4	2	2	5	1	7	3	3	5	3	2	2	3	Delhi/CAS
60	DRD176	2	4	2	2	3	5	4	4	2	2	4	4	2	2	4	1	8	3	3	4	3	8	4	3	Delhi/CAS
61	DRD177	2	4	2	2	3	6	4	4	2	2	4	4	2	2	5	1	7	3	3	5	3	8	4	2	Delhi/CAS
62	DRD179	2	3	2	2	2	6	4	4	2	3	4	4	1	2	5	1	7	3	3	5	3	2	2	2	Delhi/CAS
63	DRD181	2	3	2	2	3	6	2	4	2	2	4	4	2	2	5	1	7	3	3	5	3	2	4	3	Delhi/CAS
64	DRD186	2	3	2	2	3	6	2	4	2	7	4	4	2	2	4	1	7	3	3	5	3	7	3	2	Delhi/CAS
65	DRD19	2	4	2	2	3	5	4	4	2	2	4	4	2	2	4	1	8	3	3	4	3	8	4	3	Delhi/CAS
66	DRD192	2	3	2	2	3	6	4	4	2	2	4	4	2	2	5	1	5	3	3	5	3	6	4	3	Delhi/CAS
67	DRD197	2	3	2	2	3	6	2	4	2	2	4	4	2	1	5	1	7	3	3	5	3	2	2	3	Delhi/CAS
68	DRD199	2	3	2	2	3	5	2	4	2	7	4	4	2	2	5	1	7	3	3	5	3	8	3	2	Delhi/CAS
69	DRD2	2	4	2	2	3	5	4	4	2	2	4	4	2	2	4	1	8	3	3	4	3	8	4	3	Delhi/CAS
70	DRD20	2	3	2	2	3	4	4	4	2	2	4	4	2	2	5	1	7	3	3	4	3	2	4	2	Delhi/CAS
71	DRD201	2	3	2	2	3	6	2	4	2	7	4	4	2	2	4	1	4	3	3	5	3	8	3	2	Delhi/CAS
72	DRD204	2	3	2	2	3	6	4	4	2	2	4	4	2	2	5	1	5	3	3	5	2	6	4	3	Delhi/CAS
73	DRD206	2	3	2	2	3	5	4	4	2	2	4	4	2	2	5	1	5	3	3	5	4	7	4	3	Delhi/CAS
74	DRD209	2	4	2	2	3	6	4	4	2	2	4	4	2	2	5	1	7	3	3	5	3	8	4	2	Delhi/CAS
75	DRD211	2	4	2	2	3	5	4	4	2	2	4	4	2	2	4	1	8	3	3	4	3	8	4	3	Delhi/CAS
76	DRD212	2	3	2	2	3	6	2	4	2	7	4	4	2	2	5	1	7	3	3	5	3	7	3	2	Delhi/CAS
77	DRD213	2	3	2	2	3	5	2	4	2	7	4	4	2	2	4	1	4	3	3	5	3	8	3	2	Delhi/CAS
78	DRD214	2	3	2	2	3	6	2	4	2	7	4	4	2	2	5	1	4	3	3	5	3	8	3	2	Delhi/CAS
79	DRD215	2	3	2	2	3	6	3	4	2	2	4	4	2	2	4	1	7	3	3	5	3	2	2	3	Delhi/CAS

80	DRD217	2	3	2	2	3	6	4	4	2	2	4	4	2	2	5	1	5	3	3	5	3	5	4	3	Delhi/CAS
81	DRD22	2	4	2	2	3	6	4	4	2	2	4	4	2	2	4	1	8	3	3	4	3	8	4	3	Delhi/CAS
82	DRD221	2	3	2	2	3	5	2	4	2	7	4	4	2	2	4	1	7	3	3	5	3	8	3	2	Delhi/CAS
83	DRD230	2	4	2	2	3	5	4	4	2	2	4	4	2	2	4	1	8	3	3	4	3	8	4	3	Delhi/CAS
84	DRD231	2	4	2	2	3	6	4	4	2	2	4	4	2	2	4	1	7	3	3	3	3	8	4	3	Delhi/CAS
85	DRD232	2	4	2	2	3	5	4	4	2	2	3	4	2	2	4	1	8	3	3	4	3	7	4	3	Delhi/CAS
86	DRD234	2	4	2	2	3	6	4	4	2	2	4	4	2	2	4	1	8	3	3	4	3	8	4	3	Delhi/CAS
87	DRD238	2	3	2	2	3	6	2	4	2	2	4	4	1	2	5	1	7	3	3	5	3	2	2	3	Delhi/CAS
88	DRD239	2	4	2	2	3	5	4	4	2	2	4	4	3	2	4	1	8	3	3	3	3	8	4	3	Delhi/CAS
89	DRD240	2	3	2	2	3	6	2	4	2	2	4	4	2	2	5	1	7	3	3	5	3	2	2	3	Delhi/CAS
90	DRD243	2	2	2	2	3	6	2	4	2	7	4	4	2	2	5	1	3	3	3	5	3	8	3	2	Delhi/CAS
91	DRD244	2	4	2	2	3	5	4	4	2	2	3	4	2	2	4	1	7	3	3	4	3	8	4	3	Delhi/CAS
92	DRD250	2	3	2	2	3	6	2	4	2	7	4	4	2	2	5	1	7	3	3	5	3	7	3	2	Delhi/CAS
93	DRD252	2	3	4	2	3	4	3	4	2	3	3	4	2	2	5	1	7	3	3	5	3	8	4	3	Delhi/CAS
94	DRD255	2	4	2	2	3	5	4	4	2	2	4	4	2	2	4	1	8	3	3	3	3	8	4	3	Delhi/CAS
95	DRD263	2	4	2	2	3	5	4	4	2	2	4	4	2	2	4	1	7	3	3	4	3	8	4	3	Delhi/CAS
96	DRD267	2	3	2	2	3	6	3	4	2	2	4	4	2	2	5	1	7	3	3	5	3	2	2	3	Delhi/CAS
97	DRD281	2	3	2	2	3	5	4	4	2	2	3	4	2	2	4	1	8	3	3	4	3	7	4	3	Delhi/CAS
98	DRD284	2	3	2	2	3	5	2	4	2	7	4	4	2	2	6	1	3	3	3	5	3	8	3	2	Delhi/CAS
99	DRD288	2	4	2	2	3	6	4	4	2	2	4	4	2	2	4	1	8	3	3	4	3	8	4	3	Delhi/CAS
100	DRD296	2	3	2	2	3	6	2	4	2	7	4	4	2	2	5	1	7	3	3	5	3	7	2	2	Delhi/CAS
101	DRD299	2	5	2	2	3	3	4	4	2	2	4	4	2	2	5	1	7	3	1	5	3	8	4	2	Delhi/CAS
102	DRD304	2	3	2	2	3	5	4	4	2	2	4	4	2	2	5	1	7	3	3	5	4	1	4	3	Delhi/CAS
103	DRD314	2	5	4	2	3	5	4	4	2	6	4	4	2	2	5	1	7	3	3	5	3	8	4	3	Delhi/CAS
104	DRD316	2	3	2	2	3	5	2	4	2	7	4	4	2	2	5	1	7	3	3	5	3	8	3	2	Delhi/CAS
105	DRD317	2	3	2	2	3	6	2	4	2	7	4	4	2	2	5	1	7	3	3	5	3	7	3	2	Delhi/CAS
106	DRD318	2	3	3	2	3	6	3	4	2	2	4	4	2	2	4	1	7	3	3	5	4	8	2	3	Delhi/CAS
107	DRD321	2	4	3	2	3	6	4	4	2	5	3	4	4	2	5	1	7	3	3	5	4	7	2	2	Delhi/CAS
108	DRD323	2	3	2	2	3	6	2	4	2	7	4	4	2	2	6	1	3	3	3	5	3	8	3	2	Delhi/CAS
109	DRD325	2	4	2	2	3	6	3	6	2	2	4	4	2	2	5	1	6	3	4	5	3	6	4	3	Delhi/CAS
110	DRD326	2	3	2	2	3	5	2	4	2	7	4	4	2	2	5	1	7	3	3	5	3	8	3	2	Delhi/CAS
111	DRD331	2	3	2	2	3	6	2	4	2	2	4	4	2	2	5	1	7	3	3	5	3	2	4	3	Delhi/CAS
112	DRD333	2	3	2	2	3	6	2	4	2	7	4	4	2	2	5	1	7	3	3	5	3	8	3	2	Delhi/CAS
113	DRD338	2	3	2	2	3	6	2	4	2	7	4	4	2	2	6	1	3	3	3	5	3	8	2	2	Delhi/CAS
114	DRD344	2	3	2	2	3	5	2	4	2	7	4	4	2	2	5	1	7	3	3	5	3	7	3	2	Delhi/CAS
115	DRD348	2	3	2	2	3	6	2	4	2	2	4	4	2	2	5	1	7	3	3	5	3	2	4	3	Delhi/CAS
116	DRD358	2	3	2	2	3	6	2	4	2	7	4	4	2	2	5	1	4	3	3	5	1	8	3	2	Delhi/CAS
117	DRD361	2	3	2	2	3	6	2	4	2	7	4	4	2	2	5	1	5	3	3	5	3	8	3	2	Delhi/CAS
118	DRD37	2	4	2	2	3	4	4	4	2	2	4	4	2	2	5	1	7	3	3	5	4	6	4	3	Delhi/CAS
119	DRD370	2	3	2	2	3	6	2	4	2	2	4	4	2	2	5	1	7	3	3	5	3	2	4	3	Delhi/CAS

120	DRD373	2	3	2	2	3	5	2	4	2	7	4	4	2	2	6	1	7	3	3	5	3	8	3	2	Delhi/CAS
121	DRD378	2	3	2	2	3	6	2	4	2	2	4	4	2	2	5	1	7	3	3	5	3	2	2	3	Delhi/CAS
122	DRD38	2	3	2	2	3	6	2	4	2	2	4	4	2	2	5	1	7	3	3	5	3	2	4	3	Delhi/CAS
123	DRD385	2	3	2	2	3	6	2	4	2	7	4	4	2	2	5	1	4	3	3	5	3	7	3	2	Delhi/CAS
124	DRD389	2	3	2	2	3	6	2	4	2	2	4	4	2	2	5	1	7	3	3	5	3	2	4	3	Delhi/CAS
125	DRD39	2	4	2	1	3	7	4	4	2	2	4	4	2	2	5	1	7	3	3	5	3	5	4	3	Delhi/CAS
126	DRD394	2	3	2	2	3	6	2	4	2	7	4	4	2	2	5	1	3	3	3	5	3	8	3	2	Delhi/CAS
127	DRD402	2	2	2	2	3	6	2	4	2	2	4	4	2	2	5	1	7	3	3	5	3	2	2	3	Delhi/CAS
128	DRD404	2	3	2	2	3	5	2	4	2	7	4	4	2	2	5	1	7	3	3	5	3	8	3	2	Delhi/CAS
129	DRD407	2	4	1	2	3	5	4	4	2	2	4	4	2	2	4	1	8	3	3	3	3	8	4	3	Delhi/CAS
130	DRD408	2	3	4	2	3	3	2	4	2	6	4	4	2	2	5	1	4	3	3	5	3	8	3	3	Delhi/CAS
131	DRD410	2	3	2	2	3	6	2	4	2	7	4	4	2	2	6	1	7	3	3	5	3	7	2	2	Delhi/CAS
132	DRD413	2	4	2	2	3	5	4	4	2	2	4	4	2	2	4	1	8	3	3	4	3	8	4	3	Delhi/CAS
133	DRD437	2	3	2	2	3	6	2	3	2	2	4	4	2	1	5	1	7	3	3	5	3	2	2	3	Delhi/CAS
134	DRD438	2	3	2	2	3	6	2	4	2	7	4	4	2	2	5	1	7	3	3	5	1	8	3	2	Delhi/CAS
135	DRD442	2	3	2	2	3	6	3	4	2	2	4	4	2	2	5	1	7	3	3	5	3	2	2	3	Delhi/CAS
136	DRD444	2	3	2	2	3	5	2	3	2	2	4	4	2	2	5	1	7	3	3	5	3	2	2	3	Delhi/CAS
137	DRD450	2	4	2	2	3	6	4	4	2	2	4	4	2	2	4	1	7	3	3	4	3	8	4	3	Delhi/CAS
138	DRD451	2	3	2	2	3	6	2	4	2	2	4	4	2	1	5	1	7	3	3	4	3	5	2	3	Delhi/CAS
139	DRD457	2	5	2	2	3	5	2	4	2	2	4	4	2	2	6	1	7	3	3	5	2	8	3	2	Delhi/CAS
140	DRD46	2	3	2	2	3	5	2	4	2	2	4	4	2	2	5	1	7	3	3	5	3	2	2	3	Delhi/CAS
141	DRD466	2	3	2	2	3	5	2	4	2	7	4	4	2	2	5	1	3	3	3	5	3	8	3	2	Delhi/CAS
142	DRD471	2	3	2	2	3	6	2	4	2	7	4	4	2	2	5	1	7	3	3	5	3	7	3	2	Delhi/CAS
143	DRD478	2	3	2	2	4	5	2	4	2	2	4	4	2	2	5	1	7	3	3	4	3	5	2	3	Delhi/CAS
144	DRD479	2	4	2	2	3	6	4	4	2	2	4	4	4	2	6	1	7	3	3	4	4	4	4	2	Delhi/CAS
145	DRD48	2	3	2	2	3	6	2	3	2	2	4	4	2	2	5	1	7	3	3	5	3	2	2	3	Delhi/CAS
146	DRD486	2	3	2	2	3	6	2	4	2	2	4	4	2	2	5	1	7	3	3	5	3	2	2	3	Delhi/CAS
147	DRD489	2	3	2	2	3	5	2	4	2	7	4	4	2	2	5	1	4	3	3	5	3	8	3	2	Delhi/CAS
148	DRD490	2	3	2	2	4	6	2	4	2	2	4	4	2	2	5	1	7	3	3	5	3	2	4	3	Delhi/CAS
149	DRD491	2	5	2	2	3	5	4	4	2	6	4	4	2	2	5	1	4	3	3	5	3	7	4	3	Delhi/CAS
150	DRD496	2	3	2	2	3	6	2	4	2	2	4	4	2	1	5	1	7	2	3	5	3	5	2	3	Delhi/CAS
151	DRD50	2	4	2	2	3	6	4	4	2	2	3	4	2	2	4	1	7	3	3	4	3	8	4	3	Delhi/CAS
152	DRD501	2	3	2	2	3	6	2	4	2	2	4	4	2	1	5	1	7	3	3	5	3	2	2	2	Delhi/CAS
153	DRD503	2	3	2	2	4	6	2	4	2	2	4	4	2	2	5	1	7	3	3	5	3	2	4	3	Delhi/CAS
154	DRD514	2	4	2	2	3	6	4	4	2	2	4	4	2	1	4	2	8	3	3	3	3	7	4	3	Delhi/CAS
155	DRD521	2	4	2	2	3	5	4	4	2	2	3	4	2	2	4	1	8	3	3	4	3	8	4	3	Delhi/CAS
156	DRD522	2	5	2	2	3	3	3	4	2	6	4	4	1	2	5	1	4	3	3	5	3	8	4	3	Delhi/CAS
157	DRD524	2	3	2	2	3	6	2	4	2	2	4	4	2	2	5	1	7	3	3	3	3	2	2	3	Delhi/CAS
158	DRD532	2	5	3	2	3	3	3	4	2	6	4	4	1	1	5	1	5	3	3	5	3	8	4	3	Delhi/CAS
159	DRD545	2	4	2	2	3	4	3	4	2	2	4	4	2	2	6	1	4	3	3	3	3	7	2	2	Delhi/CAS

160	DRD552	2	3	2	2	3	6	2	4	2	2	4	4	2	2	5	1	7	2	3	5	3	5	2	3	Delhi/CAS
161	DRD566	2	3	2	2	3	6	2	4	2	2	4	4	2	1	4	1	7	3	3	5	3	2	2	2	Delhi/CAS
162	DRD577	2	4	2	2	3	5	4	4	2	2	4	4	2	2	4	1	8	3	3	3	3	7	4	3	Delhi/CAS
163	DRD578	2	5	2	2	3	5	2	4	2	2	4	4	2	2	6	1	7	3	3	5	2	8	3	2	Delhi/CAS
164	DRD590	2	3	2	2	3	6	2	4	2	1	4	4	2	2	5	1	7	3	3	5	3	2	2	3	Delhi/CAS
165	DRD592	2	5	2	2	3	5	4	4	2	6	4	4	2	2	5	1	7	3	3	5	3	8	4	3	Delhi/CAS
166	DRD593	2	5	2	2	3	5	2	4	2	2	4	4	2	2	6	1	7	3	3	5	2	7	3	2	Delhi/CAS
167	DRD597	2	4	2	2	5	6	4	4	2	2	4	4	2	2	5	1	5	3	3	4	3	7	5	2	Delhi/CAS
168	DRD600	2	5	2	2	3	5	4		2	2	4	4	2	2	5	1	7	3	3	4	3	1	2	3	Delhi/CAS
169	DRD605	2	3	2	2	3	5	2	4	2	7	4	4	1	2	5	1	6	3	3	5	3	8	3	2	Delhi/CAS
170	DRD608	2	3	2	2	3	6	2	4	2	7	4	4	2	2	6	1	7	3	3	5	3	7	3	2	Delhi/CAS
171	DRD615	2	3	2	2	3	6	2	4	2	7	4	4	1	2	5	1	7	3	3	5	3	8	3	2	Delhi/CAS
172	DRD627	2	3	2	2	3	6	2	4	2	2	4	4	2	2	5	1	7	3	3	4	3	2	3	3	Delhi/CAS
173	DRD632	2	2	2	2	3	5	2	4	2	7	4	4	2	2	6	1	7	3	3	5	3	8	3	2	Delhi/CAS
174	DRD640	2	3	2	2	3	6	2	4	2	7	4	4	2	2	5	1	7	3	3	5	3	7	3	2	Delhi/CAS
175	DRD641	2	3	2	2	4	5	2	4	2	7	4	4	2	2	5	1	7	3	3	5	3	8	3	2	Delhi/CAS
176	DRD645	2	3	2	2	3	6	2	4	2	2	4	4	2	2	5	1	7	3	3	5	3	5	4	3	Delhi/CAS
177	DRD648	2	3	2	2	3	5	2	4	2	7	4	4	2	2	5	1	7	3	3	5	3	7	3	2	Delhi/CAS
178	DRD651	2	5	2	2	3	5	2	4	2	2	4	4	2	2	6	1	7	3	3	5	2	8	3	2	Delhi/CAS
179	DRD655	2	3	2	2	3	6	2	4	2	7	4	4	2	2	5	1	7	3	3	5	3	8	3	2	Delhi/CAS
180	DRD661	2	4	2	2	3	5	4	4	2	2	4	4	2	2	4	1	8	3	3	4	3	8	4	3	Delhi/CAS
181	DRD666	2	4	2	2	3	5	4	4	2	2	4	4	2	2	4	1	8	3	3	4	3	8	4	3	Delhi/CAS
182	DRD67	2	3	2	2	3	6	2	4	2	2	4	4	2	2	5	1	7	3	3	5	3	2	4	3	Delhi/CAS
183	DRD671	2	3	2	2	3	5	2	4	2	7	4	4	1	2	5	1	7	3	3	5	3	8	3	2	Delhi/CAS
184	DRD673	2	3	2	2	4	6	2	4	2	7	4	4	2	2	5	1	7	3	3	5	3	7	3	2	Delhi/CAS
185	DRD679	2	3	2	2	3	6	2	4	2	2	4	4	2	2	5	1	7	3	3	4	2	2	2	3	Delhi/CAS
186	DRD684	2	3	2	2	4	5	2	4	2	7	4	4	2	2	6	1	7	3	3	5	3	8	3	2	Delhi/CAS
187	DRD691	2	3	2	2	3	6	2	4	2	7	4	4	1	2	5	1	7	3	3	5	3	7	3	2	Delhi/CAS
188	DRD694	2	3	2	2	3	5	2	4	2	7	4	4	3	2	5	1	7	3	3	5	3	7	3	2	Delhi/CAS
189	DRD695	2	3	2	2	3	6	2	4	2	7	4	4	2	2	5	1	7	3	3	5	3	8	3	2	Delhi/CAS
190	DRD698	2	3	2	2	3	6	2	4	2	2	4	4	2	2	5	1	7	3	3	5	3	2	4	3	Delhi/CAS
191	DRD7	2	3	2	2	3	6	2	3	2	2	4	4	2	2	5	1	7	3	3	5	3	5	2	3	Delhi/CAS
192	DRD73	2	4	2	2	3	5	4	4	2	2	4	4	2	2	4	1	8	3	3	4	3	8	4	3	Delhi/CAS
193	DRD75	2	3	2	2	3	6	2	4	2	2	4	4	2	2	5	1	7	2	3	5	3	2	2	3	Delhi/CAS
194	DRD81	2	5	2	2	3	5	2	4	2	2	4	4	2	2	6	1	7	3	3	5	2	8	3	2	Delhi/CAS
195	DRD83	2	3	2	2	3	6	2	4	2	2	4	4	2	2	5	1	7	3	3	5	3	5	4	3	Delhi/CAS
196	DRD9	2	3	2	2	3	6	2	3	2	2	4	4	2	2	5	1	7	2	3	5	3	2	2	3	Delhi/CAS
197	DRD1	2	2	4	5	3	4	2	5	2	2	7	3	2	5	5	2	2	3	3	4	4	5	1	3	EAI
198	DRD106	2	1	4	5	2	4	9	9	2	8	4	3	2	6	6	2	2	3	3	4	2	7	1	3	EAI
199	DRD107	2	1	4	5	2	4	9	a	2	9	4	3	2	6	6	2	2	3	3	4	2	7	1	3	EAI

200	DRD109	2	2	4	5	3	4	3	5	2	3	4	3	1	5	6	2	2	3	3	6	4	5	1	2	EAI
201	DRD117	2	2	4	5	4	4	3	6	2	3	6	3	1	1	6	2	2	3	3	4	4	6	1	3	EAI
202	DRD118	2	2	4	5	4	4	3	6	2	3	6	3	1	1	4	2	2	3	3	4	4	5	1	3	EAI
203	DRD12	2	2	4	5	3	4	3	5	2	2	6	3	1	4	6	2	2	3	3	5	4	6	1	3	EAI
204	DRD121	2	2	4	5	3	4	3	5	2	2	7	3	2	6	5	2	2	3	3	4	4	5	1	3	EAI
205	DRD126	2	1	4	5	2	4	9	9	2	8	4	3	2	6	6	2	2	3	3	4	2	7	1	3	EAI
206	DRD13	2	1	4	5	2	4	9	9	2	9	4	3	2	6	6	2	2	3	3	4	2	7	1	3	EAI
207	DRD135	2	2	4	5	3	4	3	5	2	3	8	3	1	3	7	2	2	3	3	6	5	5	1	3	EAI
208	DRD138	2	1	4	3	2	4	3	3	2	3	4	3	2	6	6	2	2	3	3	4	2	1	1	3	EAI
209	DRD148	2	1	4	5	2	4	3	9	2	9	6	3	2	6	6	2	2	3	3	3	2	7	1	3	EAI
210	DRD150	2	2	4	5	3	4	3	5	2	3	4	3	1	3	6	2	2	3	3	6	4	5	1	3	EAI
211	DRD151	2	1	4	5	2	4	9	a	2	8	4	3	2	6	6	2	2	3	3	4	2	7	1	3	EAI
212	DRD152	2	1	4	5	2	4	3	9	2	9	8	3	2	3	7	1	2	3	3	3	2	7	1	3	EAI
213	DRD16	2	2	2	5	3	4	3	7	2	3	4	3	2	2	6	2	2	3	3	5	4	4	1	1	EAI
214	DRD175	2	1	4	5	2	4	9	9	2	8	4	3	2	6	6	2	2	3	3	4	2	7	1	3	EAI
215	DRD18	2	1	4	5	2	4	9	a	2	9	4	3	2	6	6	2	2	3	3	4	2	7	1	3	EAI
216	DRD183	2	1	4	8	2	4	3	a	2	9	4	3	2	5	4	2	2	3	3	3	2	7	1	3	EAI
217	DRD187	2	2	4	5	2	4	3	4	2	4	9	3	2	4	7	2	3	3	3	5	7	6	1	3	EAI
218	DRD188	2	2	4	4	4	4	3	6	2	2	6	3	1	2	6	2	1	3	3	5	4	4	1	3	EAI
219	DRD189	2	2	4	4	3	4	1	5	2	2	7	3	3	5	5	1	2	3	3	5	2	6	1	2	EAI
220	DRD190	2	2	4	5	3	4	3	5	2	3	4	3	1	3	6	2	2	3	3	6	5	5	1	2	EAI
221	DRD191	2	2	4	5	3	4	3	5	2	2	7	3	2	5	5	2	2	3	3	4	4	5	1	3	EAI
222	DRD194	2	2	4	5	3	4	2	5	2	2	7	3	2	5	5	2	2	3	3	4	4	5	1	3	EAI
223	DRD195	2	2	4	5	3	4	2	5	2	2	7	3	2	5	5	2	2	3	3	4	4	5	1	3	EAI
224	DRD198	2	2	4	5	4	4	3	6	2	3	6	3	1	1	4	2	2	3	3	4	4	5	1	3	EAI
225	DRD200	2	1	4	5	2	4	3	5	2	8	4	3	2	6	6	2	2	3	3	4	2	7	1	2	EAI
226	DRD210	2	2	4	5	3	4	3	6	2	3	6	3	1	1	6	2	2	3	3	6	6	5	1	2	EAI
227	DRD216	2	2	4	4	3	4	3	5	2	2	6	3	1	4	6	1	2	3	3	5	4	6	1	3	EAI
228	DRD219	2	1	4	5	2	4	3	5	2	8	4	3	2	6	6	2	2	3	3	4	2	7	1	2	EAI
229	DRD220	2	2	4	5	2	4	3	4	2	2	9	3	2	4	7	2	3	3	3	5	7	6	1	3	EAI
230	DRD222	2	1	4	5	2	4	3	5	2	8	4	3	2	6	6	2	2	3	3	4	2	7	1	2	EAI
231	DRD224	2	2	2	5	3	4	3	a	2	6	9	3	2	4	6	2	2	3	3	4	5	4	1	1	EAI
232	DRD229	2	1	4	5	2	4	3	5	2	8	4	3	2	6	6	2	2	3	3	4	2	7	1	2	EAI
233	DRD235	2	2	4	5	4	4	3	6	2	3	6	3	1	1	4	2	2	3	3	4	4	5	1	3	EAI
234	DRD241	2	2	2	5	4	4	3	6	2	3	6	3	1	1	4	2	2	3	3	4	4	5	1	3	EAI
235	DRD245	2	1	4	5	2	4	3	5	2	8	4	3	2	6	6	2	2	3	3	4	2	7	1	2	EAI
236	DRD247	2	1	4	5	2	4	3	5	2	8	4	3	2	6	6	2	2	3	3	4	2	7	1	2	EAI
237	DRD253	2	1	4	5	2	4	3	5	2	8	4	3	2	6	6	2	2	3	3	4	2	7	1	2	EAI
238	DRD257	2	2	4	4	3	4	3	8	2	3	6	3	1	3	6	2	2	3	2	6	2	6	1	3	EAI
239	DRD258	2	1	4	5	2	4	9	9	2	8	4	3	2	6	6	2	2	3	3	4	2	7	1	3	EAI

240	DRD259	2	1	4	5	2	4	3	5	2	8	4	3	2	6	6	2	2	3	3	4	2	7	1	2	EAI
241	DRD26	2	1	4	5	2	3	3	a	2	9	4	3	2	6	6	2	2	3	3	4	2	7	1	3	EAI
242	DRD273	2	1	4	5	2	4	3	5	2	8	4	3	2	6	6	2	2	3	3	4	2	7	1	2	EAI
243	DRD274	2	1	4	5	2	4	3	5	2	8	4	3	2	6	6	2	2	3	3	4	2	7	1	2	EAI
244	DRD275	2	1	4	5	2	4	3	5	2	8	4	3	2	6	6	2	2	3	3	4	2	7	1	2	EAI
245	DRD278	2	1	4	5	2	4	3	5	2	8	4	3	2	6	6	2	2	3	3	4	2	7	1	2	EAI
246	DRD279	2	1	4	5	2	4	3	5	2	8	4	3	2	6	6	2	2	3	3	4	2	7	1	2	EAI
247	DRD290	2	2	4	5	3	4	3	6	2	3	6	3	1	1	4	2	2	3	3	4	4	5	1	3	EAI
248	DRD291	2	1	4	5	2	4	3	5	2	8	4	3	2	6	6	2	2	3	3	4	2	7	1	2	EAI
249	DRD293	2	1	4	5	2	4	3	5	2	8	4	3	2	6	6	2	2	3	3	4	2	7	1	2	EAI
250	DRD300	2	1	4	5	2	4	3	5	2	8	4	3	2	6	6	2	2	3	3	4	2	7	1	2	EAI
251	DRD306	2	2	4	5	3	4	3	6	2	3	6	3	1	1	4	2	2	3	3	6	4	5	1	3	EAI
252	DRD307	2	3	4	5	2	4	3	6	2	5	6	3	1	4	6	2	2	3	3	5	2	6	1	3	EAI
253	DRD312	2	1	4	5	2	4	3	5	2	8	4	3	2	6	6	2	2	3	3	4	2	7	1	2	EAI
254	DRD324	2	2	4	5	4	4	3	6	2	3	6	3	1	1	6	2	2	3	3	4	4	5	1	2	EAI
255	DRD330	2	1	4	5	2	4	3	5	2	8	4	3	2	6	6	2	2	3	3	4	2	7	1	2	EAI
256	DRD34	2	2	4	5	3	4	2	5	1	2	7	3	3	5	1	2	2	3	3	5	6	6	1	3	EAI
257	DRD342	2	2	4	5	3	4	3	6	2	3	6	3	1	1	4	2	2	3	3	6	6	5	1	3	EAI
258	DRD345	2	2	4	5	4	4	3	6	2	2	6	3	1	1	7	2	2	3	3	4	4	6	1	3	EAI
259	DRD346	2	2	2	5	3	4	3	9	2	8	7	3	2	2	7	2	2	3	3	6	2	4	1	1	EAI
260	DRD347	2	1	4	5	2	4	3	5	2	8	4	3	2	6	6	2	2	3	3	4	2	7	1	2	EAI
261	DRD356	2	3	4	5	2	4	3	6	2	5	6	3	1	4	6	2	2	3	3	5	2	6	1	3	EAI
262	DRD36	2	1	4	5	2	4	9	a	2	9	4	3	2	6	6	2	2	3	3	4	2	7	1	3	EAI
263	DRD360	2	3	2	5	3	5	3	8	2	2	9	3	2	4	6	2	2	3	3	5	5	4	1	1	EAI
264	DRD362	2	3	4	5	3	4	3	6	2	5	6	3	2	4	6	2	2	3	3	5	2	6	1	3	EAI
265	DRD374	2	1	4	5	2	4	3	5	2	8	4	3	2	6	6	2	2	3	3	4	2	7	1	2	EAI
266	DRD375	2	2	4	5	4	4	3	6	2	3	6	3	2	1	6	2	2	3	3	4	3	6	5	3	EAI
267	DRD387	2	1	4	5	2	4	3	5	2	8	4	3	2	6	6	2	2	3	3	4	2	7	1	2	EAI
268	DRD388	2	2	4	5	4	4	3	6	2	3	6	3	1	1	6	2	2	3	3	4	6	5	1	3	EAI
269	DRD390	2	2	4	5	4	4	3	6	2	3	6	3	1	1	6	2	2	3	3	4	4	5	1	3	EAI
270	DRD392	2	2	4	6	4	4	3	6	2	3	6	3	1	1	6	2	2	3	3	4	1	6	1	3	EAI
271	DRD395	2	2	4	7	2	4	2	4	2	4	5	3	2	6	6	2	2	3	3	5	2	7	1	2	EAI
272	DRD398	2	1	4	5	2	4	3	a	2	9	4	3	2	5	6	2	2	3	3	3	2	7	2	2	EAI
273	DRD4	2	2	4	3	3	4	2	6	2	6	9	3	2	5	5	2	2	1	3	5	4	1	1	3	EAI
274	DRD406	2	2	4	5	3	4	3	4	2	2	7	3	2	5	5	2	2	3	3	4	4	5	1	3	EAI
275	DRD419	2	2	4	4	2	5	3	6	2	9	8	3	2	4	6	2	2	1	3	4	4	5	1	3	EAI
276	DRD420	2	2	4	5	3	4	3	5	2	2	7	3	2	5	5	2	2	3	3	4	4	5	1	3	EAI
277	DRD424	2	2	4	5	4	4	3	6	2	3	6	3	1	1	4	2	2	3	3	6	4	5	1	3	EAI
278	DRD43	2	1	4	5	2	3	3	9	2	6	4	3	2	6	6	2	2	3	3	4	2	7	1	2	EAI
279	DRD433	2	1	4	5	2	4	9	a	2	9	4	3	2	6	6	2	2	3	3	4	2	7	1	3	EAI

280	DRD436	2	2	4	6	4	4	3	6	2	3	6	3	1	1	6	2	2	3	3	4	4	6	1	3	EAI
281	DRD439	2	2	4	5	3	4	2	5	2	2	7	3	2	5	5	2	2	3	3	4	4	5	1	3	EAI
282	DRD443	2	2	4	5	3	4	3	5	2	2	7	3	2	5	5	2	2	3	3	4	4	5	1	3	EAI
283	DRD448	2	2	4	4	3	4	3	5	2	2	7	3	2	5	5	2	2	3	3	4	4	5	1	3	EAI
284	DRD454	2	2	4	4	3	4	2	5	2	2	7	3	2	5	5	2	2	3	3	4	4	5	1	3	EAI
285	DRD460	2	2	4	5	3	4	3	5	2	2	7	3	2	5	5	2	2	3	3	4	4	5	1	3	EAI
286	DRD463	2	2	4	4	2	4	3	5	2	2	9	3	2	1	6	2	2	1	3	5	4	6	1	3	EAI
287	DRD467	2	2	4	5	3	4	3	6	2	3	6	3	1	1	4	2	2	3	3	4	6	5	1	3	EAI
288	DRD469	2	2	4	6	4	4	3	6	2	3	6	3	1	1	6	2	2	3	3	4	4	6	1	3	EAI
289	DRD47	2	2	4	5	3	4	3	5	2	3	4	3	1	4	6	2	2	3	3	6	4	5	1	2	EAI
290	DRD474	2	2	4	5	4	4	3	4	2	2	5	3	1	1	6	2	2	3	3	6	4	6	1	3	EAI
291	DRD485	2	2	4	5	4	4	3	6	2	3	6	3	1	1	6	2	2	3	3	6	4	5	1	2	EAI
292	DRD516	2	2	4	5	4	4	3	6	2	3	6	3	1	1	4	2	2	3	3	4	4	5	1	2	EAI
293	DRD52	2	2	4	3	3	4	3	6	2	6	9	3	2	5	5	2	2	1	3	5	4	1	1	3	EAI
294	DRD548	2	2	4	5	3	4	2	5	2	2	7	3	2	5	5	2	2	3	3	4	4	5	1	3	EAI
295	DRD549	2	2	4	5	3	4	3	5	2	2	7	3	2	5	5	2	2	3	3	4	4	5	1	3	EAI
296	DRD550	2	2	4	5	3	4	3	6	2	3	6	3	1	1	6	2	2	3	3	4	4	5	1	3	EAI
297	DRD553	2	2	4	5	4	4	3	6	2	3	6	3	1	1	4	2	2	3	3	4	4	5	1	3	EAI
298	DRD562	2	1	4	5	2	3	3	a	2	9	4	3	2	6	6	2	3	3	3	4	2	7	1	3	EAI
299	DRD57	2	2	2	5	3	4	3	6	2	3	9	3	2	4	6	2	2	3	3	5	4	3	2	1	EAI
300	DRD579	2	2	4	5	3	4	3	5	2	6	9	3	1	2	4	2	2	3	3	5	6	4	1	3	EAI
301	DRD584	2	2	4	5	3	4	3	6	2	3	6	3	2	1	4	2	2	3	3	4	4	5	1	3	EAI
302	DRD601	2	2	4	5	4	4	3	6	2	3	5	3	1	1	4	2	2	3	3	4	4	5	1	3	EAI
303	DRD603	2	2	2	5	3	4	3	9	2	5	8	3	2	4	7	2	2	3	3	5	3	4	2	1	EAI
304	DRD606	2	2	4	5	4	4	2	6	2	3	6	3	1	1	4	2	2	3	3	4	4	5	1	3	EAI
305	DRD626	2	2	4	5	4	4	2	6	2	3	5	3	1	1	4	2	2	3	3	4	4	5	1	3	EAI
306	DRD633	2	2	4	5	3	4	3	5	2	2	7	3	2	5	5	2	2	3	3	4	4	5	1	3	EAI
307	DRD657	2	2	4	5	4	4	2	4	2	2	6	3	1	1	6	2	2	3	3	4	4	7	1	3	EAI
308	DRD66	2	1	4	5	4	4	3	6	1	b	7	3	2	6	6	2	2	3	3	5	4	6	1	1	EAI
309	DRD-660	2	2	4	5	4	4	3	6	2	3	6	3	1	1	4	2	2	3	3	6	6	5	1	3	EAI
310	DRD662	2	2	4	5	3	4	3	5	2	2	7	3	2	5	5	2	2	3	3	4	4	5	1	3	EAI
311	DRD668	2	2	4	5	3	4	3	6	2	3	6	3	1	1	6	2	2	3	3	6	4	6	1	2	EAI
312	DRD669	2	2	4	5	4	3	3	6	2	3	6	3	1	1	6	2	2	3	3	4	4	5	1	3	EAI
313	DRD69	2	2	4	5	3	4	3	5	2	2	6	3	1	4	7	2	2	3	3	6	4	6	1	2	EAI
314	DRD697	2	2	4	5	3	4	3	5	2	2	7	3	2	5	5	2	2	3	3	4	4	5	1	3	EAI
315	DRD702	2	2	2	3	3	4	3	3	2	2	9	3	2	4	6	2	2	3	3	5	4	4	1	1	EAI
316	DRD71	2	2	4	4	4	4	3	6	2	3	6	3	1	1	6	2	2	3	3	4	4	4	1	3	EAI
317	DRD757	2	2	2	5	2	4	3	6	2	3	9	3	2	4	6	2	2	3	3	5	4	3	2	1	EAI
318	DRD79	2	2	2	5	3	4	3	6	2	3	9	3	2	4	6	2	2	3	3	5	4	3	1	1	EAI
319	DRD8	2	2	4	5	3	4	3	6	2	3	6	3	1	1	6	2	2	3	3	4	4	5	1	3	EAI

320	DRD82	2	2	4	4	3	4	3	5	2	2	7	3	2	5	5	2	2	3	3	4	4	5	1	3	EAI
321	DRD92	2	2	4	3	3	4	3	6	2	6	8	3	2	5	5	2	2	1	4	5	4	6	1	3	EAI
322	DRD102	2	2	3	2	5	2	3	4	2	3	3	4	4	2	5	1	4	3	3	3	3	6	1	3	Ghana
323	DRD110	2	2	3	2	5	3	3	4	2	3	3	4	4	2	5	1	4	3	3	3	3	6	1	3	Ghana
324	DRD112	2	2	3	2	5	3	3	4	2	3	3	4	4	2	5	1	4	3	3	3	3	6	1	3	Ghana
325	DRD115	2	2	3	2	5	3	3	4	2	4	3	4	4	2	5	1	4	3	3	3	3	6	1	3	Ghana
326	DRD119	2	2	3	2	5	3	3	4	2	3	3	4	4	2	5	1	4	3	3	3	3	6	1	3	Ghana
327	DRD137	2	2	3	2	5	2	3	4	2	4	3	4	4	2	4	1	4	3	3	3	3	6	2	3	Ghana
328	DRD144	2	2	3	2	5	3	3	4	3	3	3	4	4	3	5	1	4	3	3	3	3	6	1	3	Ghana
329	DRD147	2	2	5	2	5	3	3	4	2	4	3	4	4	2	5	1	4	3	3	3	3	6	1	3	Ghana
330	DRD153	2	2	3	2	5	3	3	4	2	3	3	4	4	2	5	1	4	3	3	3	3	6	1	3	Ghana
331	DRD155	2	2	3	2	5	3	3	4	2	3	3	4	4	2	5	1	4	3	3	3	3	6	1	3	Ghana
332	DRD170	2	2	3	2	5	3	3	3	2	4	3	4	4	2	5	1	4	3	3	3	3	6	1	3	Ghana
333	DRD203	2	2	3	2	5	3	3	4	2	3	2	4	4	1	5	1	4	3	3	3	3	6	1	3	Ghana
334	DRD261	2	2	3	2	5	3	3	4	2	3	3	4	4	2	5	1	4	3	3	3	4	5	2	3	Ghana
335	DRD262	2	2	3	2	5	3	3	4	2	4	3	4	4	2	5	1	4	3	3	3	3	6	1	3	Ghana
336	DRD264	2	2	2	2	5	3	3	4	2	3	3	4	4	2	5	1	3	3	3	3	3	6	1	3	Ghana
337	DRD265	2	2	3	2	5	3	3	4	2	4	3	4	4	2	5	1	4	3	3	3	3	6	1	3	Ghana
338	DRD266	2	2	3	2	5	3	3	3	2	4	3	4	4	2	5	1	4	3	3	3	3	6	1	3	Ghana
339	DRD27	2	2	3	2	5	3	3	3	2	4	3	4	4	2	5	1	4	3	3	3	4	6	1	3	Ghana
340	DRD271	2	2	3	2	5	3	3	4	2	3	3	4	4	2	5	1	4	3	3	3	3	6	1	3	Ghana
341	DRD280	2	2	3	2	5	3	2	4	2	3	3	4	4	2	5	1	4	3	3	3	3	5	1	3	Ghana
342	DRD282	2	2	3	2	5	3	3	4	2	3	3	4	4	2	5	1	4	3	3	3	3	5	1	3	Ghana
343	DRD287	2	2	3	2	5	3	3	4	2	4	3	4	4	2	5	2	4	3	3	3	3	6	1	3	Ghana
344	DRD292	2	2	3	2	5	3	3	3	2	4	3	4	4	2	5	1	4	3	3	3	3	6	1	3	Ghana
345	DRD294	2	2	3	2	5	3	3	4	2	4	3	4	4	2	5	1	4	3	3	3	3	6	1	3	Ghana
346	DRD295	2	2	3	2	5	3	3	4	2	3	2	4	4	2	5	1	4	3	3	3	4	6	1	3	Ghana
347	DRD31	3	2	3	2	5	3	3	4	2	3	3	4	4	2	5	1	4	3	3	3	3	6	2	3	Ghana
348	DRD311	2	2	3	2	5	2	3	3	2	3	3	4	3	2	5	1	4	3	3	3	3	6	1	3	Ghana
349	DRD32	2	2	3	2	5	3	3	4	2	4	3	4	4	2	5	1	4	3	3	2	3	5	1	3	Ghana
350	DRD320	2	2	3	2	5	3	3	3	2	4	3	4	4	2	5	1	4	3	3	3	3	6	1	3	Ghana
351	DRD327	2	2	3	2	5	3	3	4	2	3	3	4	4	2	5	1	4	3	3	4	3	6	1	3	Ghana
352	DRD329	2	2	3	2	5	3	2	4	2	4	3	4	4	2	5	1	4	3	3	3	3	6	2	3	Ghana
353	DRD332	2	2	3	2	5	3	3	4	2	4	3	4	4	2	5	1	4	3	3	3	4	6	1	3	Ghana
354	DRD336	2	2	3	2	5	3	2	4	2	3	3	4	3	2	5	1	4	3	3	3	3	6	1	3	Ghana
355	DRD337	2	2	3	2	5	3	3	3	2	4	3	4	4	2	5	1	4	3	3	3	3	6	1	3	Ghana
356	DRD349	2	2	3	2	5	3	2	4	2	3	3	4	4	2	5	1	4	3	3	3	3	6	2	3	Ghana
357	DRD357	2	2	3	2	5	3	2	4	2	3	3	4	4	1	5	1	4	3	3	3	3	5	1	3	Ghana
358	DRD369	2	2	3	2	3	3	3	4	2	3	3	4	4	2	5	1	4	3	3	3	3	6	1	3	Ghana
359	DRD376	2	2	3	2	5	3	3	4	2	3	3	4	4	2	5	1	4	3	3	3	3	6	1	3	Ghana

360	DRD377	2	2	3	2	5	3	2	4	2	4	3	4	4	2	5	2	4	3	3	3	3	6	1	3	Ghana
361	DRD383	2	2	3	2	5	3	3	4	2	4	3	4	4	2	5	1	4	3	3	3	3	6	1	3	Ghana
362	DRD386	2	2	3	2	5	3	3	4	2	3	3	4	4	2	5	1	3	3	3	3	3	6	1	3	Ghana
363	DRD391	2	2	3	2	5	3	3	4	2	3	3	4	4	2	5	1	4	3	3	3	3	6	1	3	Ghana
364	DRD40	2	2	3	2	5	3	3	3	2	3	3	4	4	2	5	1	4	3	3	3	3	6	4	3	Ghana
365	DRD401	2	2	3	2	5	3	3	4	2	3	3	4	4	2	5	1	4	3	3	2	3	6	1	3	Ghana
366	DRD409	2	2	3	2	5	3	3	4	2	4	3	4	4	2	5	1	4	3	3	3	3	6	1	3	Ghana
367	DRD41	2	2	3	2	3	3	2	4	2	3	3	4	4	2	5	1	4	3	3	3	3	6	1	3	Ghana
368	DRD412	2	2	3	2	5	3	3	4	2	3	3	4	4	2	5	1	4	3	3	4	3	6	1	3	Ghana
369	DRD416	2	2	3	2	5	3	3	4	2	3	3	4	4	2	5	1	4	3	3	3	3	6	1	3	Ghana
370	DRD423	2	2	3	2	5	3	3	3	2	3	3	4	4	2	5	1	4	3	3	2	3	6	1	3	Ghana
371	DRD425	2	2	3	2	5	3	3	3	2	4	3	4	4	2	5	1	4	3	3	3	3	6	1	3	Ghana
372	DRD453	2	1	3	2	5	3	2	4	2	3	3	4	4	2	5	1	4	3	3	3	3	6	1	3	Ghana
373	DRD458	2	2	3	2	3	3	3	3	2	3	3	4	4	2	5	1	4	3	3	3	3	6	1	3	Ghana
374	DRD462	3	2	3	2	5	3	2	4	2	3	3	4	4	1	5	1	4	3	3	3	3	5	2	3	Ghana
375	DRD464	2	2	3	2	5	3	2	4	2	3	3	4	4	2	5	1	4	3	3	2	3	6	1	3	Ghana
376	DRD488	2	2	3	2	5	3	3	3	2	4	3	4	4	2	5	1	4	3	3	3	3	6	1	3	Ghana
377	DRD5	2	2	3	2	5	3	3	4	2	3	3	4	4	2	5	1	4	3	3	3	3	6	1	3	Ghana
378	DRD505	2	2	3	2	5	3	3	3	2	4	3	4	4	2	5	1	4	3	3	3	3	6	1	3	Ghana
379	DRD535	2	2	3	2	5	3	3	4	2	3	3	4	4	2	5	1	4	3	3	3	3	6	1	3	Ghana
380	DRD55	2	2	3	2	5	3	3	4	2	3	3	4	4	2	5	1	4	3	3	4	3	6	1	3	Ghana
381	DRD556	2	2	3	2	5	3	3	4	2	3	3	4	4	2	5	1	4	3	3	3	3	6	1	3	Ghana
382	DRD60	2	3	3	2	5	3	3	4	2	4	3	4	4	2	5	1	3	3	3	3	3	6	1	3	Ghana
383	DRD607	2	2	3	2	5	3	3	4	2	3	3	4	4	2	5	1	4	3	3	3	3	6	1	3	Ghana
384	DRD617	2	2	3	2	5	3	1	4	2	3	3	4	4	2	5	1	4	3	3	3	3	6	1	3	Ghana
385	DRD625	2	2	3	2	5	3	3	4	2	4	3	4	4	2	4	1	4	3	3	3	3	6	1	3	Ghana
386	DRD629	2	2	3	2	5	3	3	4	2	3	3	4	4	2	5	1	4	3	3	3	3	6	1	3	Ghana
387	DRD642	2	2	3	2	5	3	2	4	2	3	3	4	4	2	5	1	4	3	3	3	3	6	1	3	Ghana
388	DRD676	2	2	3	2	5	3	3	4	2	3	3	4	4	2	5	1	4	3	3	4	3	6	1	3	Ghana
389	DRD680	2	2	3	2	5	3	3	4	2	3	3	4	4	2	5	1	4	3	3	3	3	6	1	3	Ghana
390	DRD682	2	2	3	2	5	3	3	4	2	3	3	4	4	2	5	1	4	3	3	4	3	6	1	3	Ghana
391	DRD686	2	2	3	2	5	3	2	4	2	4	3	4	4	2	5	1	4	3	3	3	3	6	2	3	Ghana
392	DRD100	2	2	3	2	3	5	2	3	2	4	3	4	4	2	5	1	5	3	3	3	3	5	3	2	Haarlem
393	DRD101	2	2	3	2	3	5	3	3	2	4	3	4	4	2	5	2	5	3	3	3	3	5	3	2	Haarlem
394	DRD103	2	2	4	2	2	6	3	3	2	4	2	4	4	2	5	1	5	3	3	3	3	5	3	2	Haarlem
395	DRD128	2	2	3	2	3	5	3	3	2	4	3	4	3	2	5	1	5	3	3	3	3	5	3	2	Haarlem
396	DRD145	2	2	3	2	3	5	2	3	2	4	3	4	4	2	5	1	5	3	3	3	3	6	3	2	Haarlem
397	DRD182	2	2	4	2	2	6	3	3	2	4	2	4	4	2	5	1	5	3	3	3	3	5	3	2	Haarlem
398	DRD242	2	2	3	2	3	5	3	3	2	4	4	4	3	2	5	1	5	3	3	3	3	5	3	2	Haarlem
399	DRD25	2	2	3	2	3	5	3	3	2	4	3	4	4	2	5	1	5	3	3	3	3	6	3	2	Haarlem

400	DRD251	2	2	4	2	2	6	3	3	2	4	2	4	4	2	5	1	5	3	3	3	3	6	3	2	Haarlem
401	DRD272	2	2	4	2	2	4	3	3	2	4	2	4	4	2	5	1	5	3	3	3	3	5	3	2	Haarlem
402	DRD286	2	1	3	2	3	5	3	3	2	6	3	4	3	2	3	1	5	3	3	3	3	5	3	2	Haarlem
403	DRD289	2	2	3	2	3	6	3	3	2	5	3	4	4	2	5	1	5	3	3	3	2	5	3	2	Haarlem
404	DRD303	2	2	3	2	3	4	2	3	2	7	3	4	4	2	5	1	5	3	3	3	3	6	3	2	Haarlem
405	DRD313	2	2	4	2	2	4	3	3	2	4	2	4	4	2	5	1	5	3	3	3	3	5	3	2	Haarlem
406	DRD319	2	2	3	2	3	5	3	3	2	3	3	4	4	2	5	2	5	3	3	3	3	5	3	2	Haarlem
407	DRD366	2	2	4	2	2	6	3	3	2	4	3	4	4	2	5	1	5	3	3	3	2	5	3	2	Haarlem
408	DRD372	2	2	4	2	3	6	3	3	2	4	2	4	4	2	5	1	5	3	3	3	3	5	3	2	Haarlem
409	DRD399	2	2	4	2	2	4	3	3	2	4	3	4	4	2	5	1	5	3	3	3	3	6	3	2	Haarlem
410	DRD403	2	2	3	2	3	5	3	3	2	3	3	4	4	2	5	2	5	3	3	3	3	5	3	2	Haarlem
411	DRD42	2	2	3	2	3	5	2	3	1	7	3	4	4	2	5	1	5	3	3	3	3	6	3	2	Haarlem
412	DRD429	2	2	3	2	3	5	3	3	2	4	3	4	4	2	5	1	5	3	3	3	3	7	3	2	Haarlem
413	DRD441	2	2	4	2	2	6	3	3	2	4	2	4	4	2	5	1	5	3	3	3	3	5	3	2	Haarlem
414	DRD445	2	2	4	2	2	6	3	3	2	4	3	4	4	2	5	1	5	3	3	3	3	5	3	2	Haarlem
415	DRD447	2	2	4	2	3	4	3	3	2	4	2	4	4	2	5	1	5	3	3	3	3	6	3	2	Haarlem
416	DRD45	2	2	3	2	3	5	3	3	2	6	3	4	4	2	4	1	5	3	3	3	3	7	3	2	Haarlem
417	DRD484	2	2	4	2	2	6	3	3	2	4	2	4	4	2	5	1	5	3	3	3	2	5	3	2	Haarlem
418	DRD49	2	2	3	2	3	5	2	3	2	3	3	4	4	2	5	1	5	3	3	3	3	5	3	2	Haarlem
419	DRD502	2	2	4	2	2	6	3	3	2	4	2	4	4	2	5	1	5	3	3	3	3	5	3	2	Haarlem
420	DRD505	2	2	4	2	2	4	3	3	2	4	2	4	4	2	5	1	5	3	3	3	3	6	3	2	Haarlem
421	DRD508	2	2	4	2	2	6	3	3	2	4	2	4	4	2	5	1	5	3	3	3	3	5	3	2	Haarlem
422	DRD517	2	2	4	2	4	5	3	3	2	1	2	4	4	2	5	1	5	3	3	3	3	5	3	2	Haarlem
423	DRD537	2	2	4	2	2	4	3	3	2	4	2	4	4	2	5	1	5	3	3	3	3	5	3	2	Haarlem
424	DRD54	2	2	4	2	2	6	3	3	2	4	3	4	4	2	5	1	5	3	3	3	3	5	3	2	Haarlem
425	DRD547	2	2	4	2	3	6	3	3	2	4	2	4	4	2	5	1	5	3	3	3	3	6	3	2	Haarlem
426	DRD561	2	2	4	2	2	6	3	3	2	4	2	4	4	2	5	1	5	3	3	3	2	5	3	2	Haarlem
427	DRD567	2	2	4	2	2	6	3	3	2	4	3	4	4	2	5	1	5	3	3	3	3	5	3	2	Haarlem
428	DRD580	2	2	4	2	2	6	3	3	2	4	2	4	4	2	5	1	5	3	3	3	3	5	3	2	Haarlem
429	DRD599	2	2	4	2	2	6	3	3	2	4	3	4	4	2	5	1	5	3	3	3	3	5	3	2	Haarlem
430	DRD623	2	2	4	2	2	6	3	3	2	4	2	4	4	2	5	1	5	3	3	3	2	5	3	2	Haarlem
431	DRD63	2	2	3	2	2	5	3	3	2	4	3	4	4	2	5	2	5	3	3	3	2	5	3	2	Haarlem
432	DRD630	2	2	3	2	3	5	3	3	2	3	3	4	4	2	5	1	5	3	3	3	3	5	3	2	Haarlem
433	DRD64	2	2	4	2	3	6	3	3	2	4	3	4	4	2	5	1	5	3	3	3	2	5	3	2	Haarlem
434	DRD672	2	2	4	2	2	6	3	3	2	4	2	4	4	2	5	1	5	3	3	3	3	5	3	2	Haarlem
435	DRD678	2	2	4	2	3	4	2	3	2	4	2	4	4	2	5	1	5	3	3	3	3	6	3	2	Haarlem
436	DRD70	2	2	4	2	2	6	3	3	2	4	3	4	4	2	5	1	5	3	3	3	3	5	3	2	Haarlem
437	DRD124	2	2	2	2	1	4	3	3	2	3	2	4	1	2	5	1	4	3	3	4	2	3	2	2	LAM
438	DRD159	2	4	4	2	1	4	2	3	2	2	2	4	1	1	6	1	4	3	5	3	3	3	2	2	LAM
439	DRD160	2	2	2	2	1	4	3	3	2	3	2	4	1	2	6	1	4	3	1	4	2	3	2	2	LAM

440	DRD168	1	3	4	2	2	4	3	3	2	2	1	4	1	2	6	1	5	3	3	2	2	5	2	2	LAM
441	DRD236	2	2	2	2	1	4	3	3	2	3	2	4	1	2	6	1	4	3	3	4	2	3	2	2	LAM
442	DRD269	2	2	2	2	1	4	3	3	2	3	2	4	1	2	6	1	4	3	3	4	2	3	2	2	LAM
443	DRD30	2	2	2	2	1	4	3	3	2	3	2	4	1	2	6	1	4	3	3	4	2	3	2	2	LAM
444	DRD315	2	4	4	2	2	4	2	3	2	3	2	4	1	1	6	1	4	3	5	3	3	8	2	2	LAM
445	DRD322	2	2	2	2	1	4	3	3	2	3	2	4	1	2	5	1	4	3	2	4	2	3	2	2	LAM
446	DRD364	2	1	2	2	1	4	3	3	2	3	2	4	1	2	6	1	4	3	3	4	2	3	2	2	LAM
447	DRD367	2	2	2	2	1	4	3	3	2	3	2	4	1	2	6	1	4	3	3	4	2	3	2	2	LAM
448	DRD396	1	3	4	2	2	4	2	3	2	2	1	4	1	2	6	1	5	3	3	2	2	5	2	2	LAM
449	DRD422	2	2	2	2	1	4	3	3	2	3	2	4	1	2	5	1	4	3	3	4	2	3	2	2	LAM
450	DRD44	2	4	4	2	1	4	2	3	2	3	2	4	1	1	6	1	4	3	5	3	3	8	2	2	LAM
451	DRD446	2	1	2	2	1	4	3	3	2	3	2	4	1	2	6	1	4	3	1	4	2	3	2	2	LAM
452	DRD449	2	2	2	2	1	4	3	3	2	3	2	4	1	2	6	1	4	3	3	4	2	3	2	2	LAM
453	DRD456	2	2	2	2	1	4	3	3	2	3	2	4	1	2	6	1	4	3	3	4	2	3	2	2	LAM
454	DRD476	2	1	2	2	1	4	3	3	2	3	2	4	1	2	6	1	4	3	3	4	2	3	2	2	LAM
455	DRD483	2	2	2	2	1	4	3	3	2	3	2	4	1	2	5	1	4	3	3	4	2	3	2	2	LAM
456	DRD495	2	2	2	2	1	4	3	3	2	3	2	4	1	2	5	1	4	3	1	4	2	3	2	2	LAM
457	DRD498	2	1	2	2	1	4	3	3	2	3	2	4	1	2	6	1	4	3	3	4	2	3	2	2	LAM
458	DRD518	2	2	2	2	1	4	3	3	2	3	2	4	1	2	6	1	4	3	3	4	2	3	2	2	LAM
459	DRD519	2	4	4	2	2	4	2	3	2	2	2	4	1	1	6	1	4	3	5	3	3	3	2	2	LAM
460	DRD53	2	2	2	2	1	4	3	3	2	3	2	4	1	2	6	1	4	3	3	4	2	3	2	2	LAM
461	DRD663	2	3	2	2	1	4	3	3	2	3	2	4	1	2	5	1	4	3	1	4	2	3	2	2	LAM
462	DRD683	2	3	2	2	1	4	3	3	2	3	2	4	1	2	6	1	4	3	3	4	2	3	2	2	LAM
463	DRD104	2	2	4	2	3	2	2	5	2	3	3	4	2	1	5	1	4	3	2	3	3	3	2	2	NEW-1
464	DRD156	2	2	4	2	3	2	2	5	2	2	3	6	2	1	5	1	4	3	2	3	3	8	2	2	NEW-1
465	DRD184	2	1	4	2	3	2	2	5	2	3	3	4	2	1	5	1	4	3	2	3	3	7	2	2	NEW-1
466	DRD340	2	2	4	2	3	2	2	5	2	2	3	4	2	1	5	1	4	3	2	3	3	3	2	2	NEW-1
467	DRD365	2	1	4	2	3	2	2	5	2	2	3	4	2	1	5	1	4	3	2	3	3	8	2	2	NEW-1
468	DRD435	2	2	4	2	3	2	2	5	2	3	3	4	1	1	5	1	4	3	2	3	3	3	2	2	NEW-1
469	DRD56	2	2	4	2	3	2	2	5	2	2	3	4	2	1	5	1	4	3	2	3	3	8	2	2	NEW-1
470	DRD58	2	1	4	2	3	2	2	5	2	3	3	4	2	1	5	1	4	3	2	3	3	3	2	2	NEW-1
471	DRD639	2	2	3	2	3	2	2	5	2	2	3	4	2	1	5	1	4	3	2	3	3	8	2	2	NEW-1
472	DRD68	2	1	4	2	3	2	2	5	2	3	3	4	2	1	5	1	4	3	2	3	3	8	2	2	NEW-1
473	DRD205	3	3	4	2	4	3	3	1	2	4	4	3	2	2	5	1	4	3	3	2	3	9	2	2	S
474	DRD21	3	3	4	3	2	3	3	1	2	5	4	3	2	2	5	1	4	3	3	2	3	7	2	2	S
475	DRD233	3	3	4	3	4	3	3	1	2	4	4	3	2	2	5	1	4	3	3	2	3	5	2	2	S
476	DRD254	3	3	4	3	4	3	3	1	2	4	4	3	2	2	5	1	4	3	3	2	3	5	2	2	S
477	DRD28	3	3	4	3	5	3	3	1	2	4	4	3	2	2	5	1	3	3	3	2	3	5	2	2	S
478	DRD308	2	3	4	3	5	3	3	1	2	5	4	4	2	2	6	1	3	3	3	3	3	4	1	2	S
479	DRD310	1	4	4	2	5	3	5	2	2	4	2	4	2	3	5	1	3	2	2	3	6	4	1	2	S

480	DRD335	3	3	4	3	4	3	3	1	2	4	4	3	2	2	5	2	4	3	3	2	3	5	4	2	S
481	DRD380	3	3	4	3	4	3	2	1	2	4	4	3	2	2	5	1	5	3	3	2	3	5	2	2	S
482	DRD382	3	3	4	3	4	3	3	1	2	4	4	3	2	2	5	1	4	3	3	2	3	5	2	2	S
483	DRD397	3	3	4	3	4	3	3	1	2	4	4	3	2	2	5	2	5	3	3	2	3	5	2	2	S
484	DRD452	3	3	4	3	4	3	3	1	2	4	4	3	2	2	5	1	4	3	3	2	3	5	2	2	S
485	DRD472	3	3	4	3	2	3	3	1	2	5	4	3	2	2	5	1	4	3	3	2	3	7	2	2	S
486	DRD523	3	3	4	3	4	3	3	1	2	4	4	3	2	2	5	1	4	3	3	2	3	5	2	2	S
487	DRD690	3	3	4	2	4	3	3	1	2	4	4	3	2	2	5	1	5	3	3	2	3	5	2	2	S
488	DRD692	3	3	4	2	4	3	3	1	2	4	4	3	2	2	4	1	5	3	3	2	3	5	2	2	S
489	DRD72	3	3	4	3	2	3	3	1	2	5	4	3	2	2	5	1	4	3	3	2	3	7	2	2	S
490	DRD225	2	4	4	1	2	5	1	2	2	2	3	3	4	2	5	1	1	3	3	3	3	6	3	2	TUR
491	DRD302	2	4	4	1	2	5	1	2	2	2	3	3	4	2	5	1	1	3	3	3	3	6	3	2	TUR
492	DRD305	2	4	4	1	2	5	1	2	2	2	3	3	4	2	5	1	1	3	3	3	3	6	3	2	TUR
493	DRD131	2	1	5	2	2	3	2	3	2	2	4	4	2	2	5	1	4	3	3	4	2	3	2	2	Ugandal
494	DRD146	2	1	5	2	3	3	2	3	2	3	4	4	2	2	5	1	4	3	3	4	2	3	2	2	Ugandal
495	DRD180	2	1	5	2	3	3	2	3	2	3	4	4	2	2	5	1	5	3	3	4	2	4	2	2	Ugandal
496	DRD23	2	1	5	2	3	3	2	3	2	2	4	4	2	2	5	1	5	3	3	4	2	4	2	2	Ugandal
497	DRD270	2	1	5	2	3	3	2	3	2	3	4	4	2	2	5	1	5	3	3	4	2	3	2	2	Ugandal
498	DRD29	2	1	5	2	2	3	2	3	2	2	4	4	2	2	5	1	5	3	3	4	2	4	2	2	Ugandal
499	DRD430	2	1	5	2	3	3	2	3	2	2	4	4	2	2	4	1	5	3	3	4	2	3	2	2	Ugandal
500	DRD434	2	1	5	2	3	3	2	3	2	2	4	4	2	2	5	1	4	3	3	4	2	4	2	2	Ugandal
501	DRD541	2	1	4	2	2	3	3	3	2	3	4	4	2	2	3	1	5	3	2	3	4	1	2	2	Ugandal
502	DRD543	2	1	5	2	3	3	2	3	2	2	4	4	2	2	5	1	4	3	3	3	2	4	2	2	Ugandal
503	DRD604	2	1	4	2	2	3	3	2	2	3	4	4	2	2	3	1	5	3	2	3	4	1	2	2	Ugandal
504	DRD656	2	2	5	2	3	3	2	3	2	2	4	4	2	2	5	1	4	3	3	4	2	4	2	2	Ugandal
505	DRD363	2	4	5	3	2	2	3	1	2	3	5	2	2	5	4	2	5	3	3	3	2	5	3	2	Undefined
506	DRD384	2	1	5	2	5	3	2	2	2	3	3	4	2	2	5	1	3	3	3	3	4	4	2	2	Undefined
507	DRD134	2	4	4	2	2	9	2	3	2	2	4	4	4	2	5	1	1	3	3	3	3	8	3	2	URAL
508	DRD277	2	4	4	2	2	9	2	3	2	2	4	4	4	2	5	1	1	3	3	3	3	8	3	2	URAL
509	DRD350	2	2	4	2	2	8	2	5	2	2	4	4	4	2	5	1	1	3	3	3	3	8	3	2	URAL
510	DRD353	2	4	4	2	2	9	2	3	2	2	4	4	4	2	5	1	1	3	3	3	3	8	3	2	URAL
511	DRD354	2	3	4	3	2	9	2	3	2	1	5	4	4	2	5	1	1	3	3	3	3	7	3	2	URAL
512	DRD368	2	3	4	3	2	9	2	3	2	1	5	4	4	2	5	1	1	3	3	3	3	8	3	2	URAL
513	DRD440	2	3	4	3	2	9	2	3	2	1	5	4	4	2	5	1	2	3	3	3	3	8	3	2	URAL
514	DRD487	2	3	4	3	2	8	2	3	2	1	5	4	4	2	5	1	1	3	3	2	3	8	3	2	URAL
515	DRD612	2	3	3	3	2	9	2	3	2	1	5	4	4	2	5	1	2	3	3	3	3	8	3	2	URAL
516	DRD613	2	4	4	2	2	9	2	3	2	2	4	4	4	2	5	1	1	3	3	3	3	8	3	2	URAL
517	DRD675	2	2	4	2	2	a	2	5	2	2	4	4	4	2	5	1	1	3	3	3	3	8	3	2	URAL
518	DRD227	2	2	3	2	5	4	3	4	2	4	3	4	4	2	5	1	5	3	3	3	6	7	3	2	X
519	DRD248	2	2	2	2	5	4	3	4	2	4	3	4	4	2	5	1	5	3	3	3	6	8	3	2	X

520	DRD355	2	2	3	2	5	4	3	4	2	4	3	4	4	2	5	1	5	3	3	3	5	8	3	2	X
521	DRD379	2	2	3	2	5	3	3	4	2	4	3	4	4	2	5	1	5	3	3	3	6	8	3	2	X
522	DRD431	2	2	3	2	4	4	3	4	2	4	3	4	4	2	5	1	5	3	3	3	6	7	3	2	X
523	DRD473	2	2	3	2	5	4	3	4	2	4	3	4	3	2	5	1	5	3	3	3	6	8	3	2	X
524	DRD6	2	2	4	2	5	3	3	4	2	4	3	4	4	2	5	1	5	3	3	3	6	9	3	2	X
